# *Acinetobacter baumannii* treatment strategies: a review of therapeutic challenges and considerations

**DOI:** 10.1128/aac.01063-24

**Published:** 2025-07-09

**Authors:** Christine J. Kubin, Christopher Garzia, Anne-Catrin Uhlemann

**Affiliations:** 1 Department of Medicine, Columbia University Vagelos College of Physicians and Surgeons 12294, New York, New York, USA; 2 Department of Pharmacy, NewYork-Presbyterian Hospital 2034, New York, New York, USA; Johns Hopkins University School of Medicine, Baltimore, Maryland, USA

**Keywords:** Acinetobacter, antimicrobial chemotherapy, pharmacokinetics

## Abstract

Antimicrobial resistance poses a major challenge in the treatment of *Acinetobacter baumannii. Acinetobacter* spp. are intrinsically resistant to a number of commonly used antibiotics. Over the past 3 years, the European and American Professional Societies have provided important guidelines on the treatment options for carbapenem-resistant *A. baumannii* (CRAB). Here, we review the recent literature on combination regimens for CRAB as well as carbapenem-susceptible *A. baumannii* infections. We discuss the strengths and weaknesses of various agents used in combination, depending on the site of infection and their pharmacokinetic properties. Consistent with the 2024 Infectious Diseases of America (IDSA) update, sulbactam-durlobactam, in combination with background carbapenem therapy, remains the combination with the greatest reduction in mortality for pulmonary infections and has promising outcomes in bloodstream infections with CRAB. Sulbactam-based combination therapy remains an ideal part of targeted strategies and has been shown to be associated with reduced mortality. Certain agents have been highlighted in the literature for suboptimal outcomes, primarily pulmonary infections treated with cefiderocol, tigecycline, and eravacycline. Studies including non-pulmonary infections, specifically bacteremia and central nervous system (CNS) infections, are overall limited to case series and subgroup analyses. Important areas for further research include breakpoint evaluations for eravacycline and minocycline as well as subclinical resistance in cefiderocol.

## INTRODUCTION

The genus Acinetobacter is diverse and has undergone many taxonomic changes over the years. The most common species causing human infections are *Acinetobacter baumannii*, *Acinetobacter calcoaceticus,* and *Acinetobacter lwoffii* (1). *A. baumannii* is part of the *Acinetobacter calcoaceticus–baumannii* complex (ACB complex), which also includes *Acinetobacter pittii*, *Acinetobacter nosocomialis, Acinetobacter calcoaceticus*, *Acinetobacter seifertii*, and *Acinetobacter dijkshoorniae* (2, 3). *A. baumannii* is the most clinically relevant of the ACB complex, being associated with increased mortality and virulence compared with non-*baumannii* ACB complex species and is also the most likely to exhibit multidrug resistance (4).

In 2017, the World Health Organization (WHO) classified carbapenem-resistant *A. baumannii* (CRAB) as a critical-priority bacterium in need of future development of antibiotics and maintained CRAB in the critical priority category in the 2024 update (5, 6). In 2019, the Centers for Disease Control and Prevention named CRAB an urgent threat, citing limited treatment options and the potential for mortality and large outbreaks among hospitalized and nursing home patients (7).

*A. baumannii* is a recognized cause of healthcare-associated infections (HAIs), including pneumonia, bacteremia, and urinary tract infections (UTIs). *Acinetobacter* spp. are known skin colonizers in 20%–25% of healthy individuals, which can increase to 75% in hospitalized patients (8). Risk factors for *A. baumannii* include prolonged hospital length of stay, intensive care unit exposure, mechanical ventilation, use of broad-spectrum antibiotics, surgery, invasive procedures, and underlying severity of illness (9–11). During the period of 2018–2021, the U.S. National Healthcare Safety Network (NHSN) surveillance data reported that *A. baumannii* was the causative pathogen for 0.4% (*n* = 1,951) of all HAIs in both adult and pediatric patients (12). Among *Acinetobacter* spp. HAIs, carbapenem resistance is a significant concern and is as high as 45% in device-associated *Acinetobacter* spp. HAIs, 45%–51% of catheter-associated urinary tract infections, 41% of intensive care unit central line-associated bloodstream infections (CLABSIs), 36% of possible ventilator-associated pneumonias, and 28% of surgical site infections. Notably, carbapenem non-susceptibility was higher in long-term acute care hospitals compared with other settings. Mortality rates in patients with *Acinetobacter* spp. infections have been reported in the range of 29%–73% and may be higher in patients presenting with septic shock (13–15). Several studies have documented increased hospital length of stay, ICU utilization, and worse outcomes in patients with multidrug-resistant or CRAB infections, including a 16% higher attributable mortality in patients with bloodstream infections associated with carbapenem resistance (16–20). In addition, CRAB infections are associated with greater mortality compared with other carbapenem-resistant organisms (13, 21–23). Increased severity of illness, the identification of carbapenem resistance, and the associated challenges with choosing optimal therapy contribute to the worse outcomes in these patients.

## CHALLENGES OF ANTIMICROBIAL RESISTANCE IN *A. BAUMANNII* AND OVERVIEW OF MECHANISMS OF RESISTANCE

Antimicrobial resistance poses a major challenge in the treatment of *A. baumannii* worldwide. *Acinetobacter* spp. are intrinsically resistant to a number of commonly used antibiotics, including aminopenicillins, first- and second-generation cephalosporins and chloramphenicol (24, 25). Global data on the prevalence of antibiotic resistance in *A. baumannii* over a 10-year period documented increases in resistance across multiple classes of drugs (26). For the 6-year period of 2011–2016, antibiotic resistance to the broad-spectrum beta-lactams ceftazidime, cefepime, and piperacillin-tazobactam was the highest among the antibiotics tested at rates of 81.8%–89.7%, 78.2%–89.4%, and 80.1%–85.2%, respectively. The most active beta-lactam was ampicillin-sulbactam with resistance reported as 72.3%-74.0% in 2011–2016. In that same 2011–2016 period, imipenem and meropenem showed further increases in resistance up to 73.9%-77.8% and 70.1%-81.1%, respectively. Resistance rates vary geographically, and only sparse data on the prevalence of resistance are available from some countries. Of particular concern are the higher reported rates of CRAB from resource-limited settings (27). In 2019, in the United States, the overall incidence of CRAB was low at 0.7 per 100,000 population, but the majority of isolates tested (72.7%) contained an acquired carbapenemase gene. The two most active antibiotics *in vitro* are sulbactam-durlobactam (97.7% to 98.3% susceptible) and cefiderocol (96.9% susceptible) (28–30).

Resistance to beta-lactam antibiotics is primarily mediated by beta-lactamases. All strains of *A. baumannii* contain a non-inducible chromosomal AmpC beta-lactamase (31, 32). Extended-spectrum beta-lactamases, including TEM, SHV, and CTX-M types, have been described (33–37). Carbapenem resistance is the most problematic and may be mediated by Ambler Class A *Klebsiella pneumoniae* carbapenemases (KPC); Ambler Class B metallo-beta-lactamases, including NDM, VIM, and IMP; and Ambler Class D OXA enzymes (33, 38, 39). OXA enzymes are the most common carbapenemases harbored by *A. baumannii,* with OXA-23 being most commonly found among clinical isolates globally at >80%, followed by OXA-40/24 and OXA-58 (40–42). Notably, OXA-51 is chromosomally encoded, whereas others are horizontally transferred via mobile genetic elements (43). In comparison to the global epidemiology, North and Latin America have higher reports of OXA-40-like-positive isolates (32%–46%) (44). OmpA is the most abundant outer membrane porin in *A. baumannii,* and several outbreaks of imipenem-resistant *A. baumannii* have been associated with this mechanism (45, 46). Resistance to aminoglycosides is often mediated through aminoglycoside-modifying enzymes, most commonly encoded by AphA6 (confers resistance to amikacin and kanamycin) and AadB (confers resistance to gentamicin, tobramycin, and kanamycin) genes, which can phosphorylate, adenylate, or acetylate the antibiotic (47). Aminoglycoside susceptibility can also be affected by mutations in the plasmid-encoded 16s RNA methylase gene target (*armA*) causing high-level resistance to gentamicin, tobramycin, amikacin, and kanamycin and may co-exist with modifying enzymes (48). Overexpression of efflux pumps can lead to decreased concentrations of antibiotics before they reach their intended target. *Acinetobacter* spp. are known to harbor six clinically relevant families of efflux pumps: the major facilitator superfamily (MFS), small multidrug resistance (SMR) family, ATP-binding cassette (ABC) family, multidrug and toxic compound extrusion (MATE) family, proteobacterial antimicrobial compound efflux (PACE) family, and the resistance-nodulation-division (RND) family, which contribute to decreased susceptibility to quinolones, macrolides, chlorhexidine, chloramphenicol, aminoglycosides, tetracyclines/tigecycline, and some beta-lactams (49). The RND family and their upregulation are most commonly associated with multidrug resistance. Quinolone resistance may also be mediated by mutations in both *gyrA* and *parC* (31). Resistance to colistin has been attributed to loss of lipopolysaccharide (LPS), modification of LPS via PetN moieties, and efflux pump systems (50). Many of the described resistance mechanisms are acquired through plasmid-mediated horizontal gene transfer, rendering *Acinetobacter* spp. resistant to multiple classes of antibiotics.

## SPECIFIC TREATMENT OPTIONS FOR CARBAPENEM-RESISTANT *A. BAUMANNII*

Considerations for the management of *A. baumannii* infection are multifactorial and complex, requiring an understanding of the strengths and limitations of clinical data, pharmacokinetic challenges, and how specific resistance mechanisms impact therapy. This section is focused on navigating current guidance with these considerations in mind.

The 2022 European Society of Clinical Microbiology and Infectious Diseases (ESCMID) treatment guidelines for multidrug-resistant gram-negative *bacilli* recommended the use of ampicillin-sulbactam for sulbactam-susceptible isolates of CRAB or a polymyxin or high-dose tigecycline for sulbactam-resistant isolates (22). Notably, cefiderocol was not recommended, as it offered no advantage to other available therapies in terms of clinical and microbiologic outcomes for CRAB (51). For severe infections, combination therapy was recommended with at least two *in vitro* active antibiotics among polymyxin, aminoglycoside, tigecycline, and sulbactam combinations. For CRAB isolates exhibiting meropenem minimum inhibitory concentrations (MICs) ≤ 8 mcg/mL, these guidelines recommended high-dose extended-infusion carbapenem. In 2023, and then with an update in 2024, the Infectious Diseases Society of America offered different recommendations for the treatment of CRAB (52, 53). In 2023, ampicillin-sulbactam at total daily doses of 6–9 g of sulbactam was recommended in combination with at least one additional agent as the preferred therapy for CRAB. A sulbactam-based regimen was endorsed for all isolates, including intermediate and resistant, due to the proposed benefit of saturating penicillin-binding proteins and the potential for inaccurate susceptibility testing. In 2024, sulbactam-durlobactam in combination with a carbapenem replaced ampicillin-sulbactam as the preferred therapy. Additionally, ampicillin-sulbactam combination therapy was moved to alternative therapy, exclusively recommending 9 g of sulbactam daily. In addition, the 2024 guidance document suggests that the additional combination agents include polymyxin B, minocycline as the preferred tetracycline derivative, or cefiderocol. Differences in recommendations between ESCMID and IDSA are likely related to available evidence at the time of each publication, as sulbactam-durlobactam was not approved for use in the USA and Europe until 2023.

### Ampicillin-sulbactam

Ampicillin-sulbactam is a beta-lactam-beta-lactamase inhibitor combination that has bactericidal effects against *A. baumannii* primarily due to the sulbactam component saturating penicillin-binding proteins 1 and 3 (PBP1 and PBP3) (54). In the United States, until recently, sulbactam was only available commercially as ampicillin-sulbactam in a 2:1 ratio. Therefore, much of the published literature reflects the use of ampicillin-sulbactam at various doses. This has changed with the introduction of sulbactam-durlobactam in a 1:1 ratio (discussed below).

The Clinical and Laboratory Standards Institute (CLSI) has published breakpoints with sulbactam MIC (based on the sulbactam component of ampicillin-sulbactam tested in a 2:1 ratio) ≤4 mcg/mL defined as susceptible, 8 mcg/mL as intermediate, and 16 mcg/mL as resistant (55). Murine infection models have established that free sulbactam concentrations above the minimum inhibitory concentration (fT >MIC) will predict efficacy (56, 57). Existing data for sulbactam against *A. baumannii* suggest bactericidal activity when a fT >MIC of >60% in a thigh infection model and fT >MIC of >40% in a lung infection model (56). These are based on data with a wild-type isolate, which achieved a 3-log kill and did not account for protein binding. More recent data suggest targeting regimens to achieve a 1-log kill for beta-lactams (57, 58). Using pharmacokinetic data from patients with ventilator-associated pneumonia, a pharmacodynamic target of 1-log kill (28.4% fT >MIC), and accounting for protein binding, sulbactam standard doses of 1 g every 6 h as a 30-min infusion (i.e., ampicillin-sulbactam 3 g every 6 h) provided >90% probability of target attainment (PTA) up to a MIC of 4 mcg/mL (57). For sulbactam-intermediate isolates, sulbactam administered at 3 g every 8 h as a 4-h infusion is needed to achieve a similar probability of target attainment. This study further suggested that a higher target of fT >MIC of 60.4% was needed for bactericidal activity against sulbactam and meropenem-resistant isolates consistent with previous data (56). The current recommended regimen of 3 g every 8 h as a 4-h infusion (i.e., ampicillin-sulbactam 9 g every 8 h) for CRAB, however, only achieved ≤57% probability of target attainment for isolates with sulbactam MICs ≥ 16 mcg/mL, highlighting the potential for undertreatment in the setting of elevated MICs. No significant adverse effects using high daily doses of sulbactam of ≥8 g/day administered as ampicillin-sulbactam have been reported. The safety of high doses of ampicillin-sulbactam has been demonstrated in previous studies (59–61). Many of these studies, however, did not contain patients with significant renal disease. In only one of these did the authors comment on sulbactam dose adjustments, specifically decreasing by 50% in patients with a creatinine clearance of <20 mL/min (59).

Although ampicillin-sulbactam non-susceptibility is common among CRAB isolates (29), there is growing evidence that sulbactam-containing regimens are associated with improved outcomes. In support of combination therapy is a previous small study that utilized high-dose ampicillin-sulbactam in carbapenem-resistant, ampicillin-sulbactam susceptible or intermediate (MIC <16 mcg/mL) strains (59). This open-label prospective study in 39 patients with *A. baumannii* ventilator-associated pneumonia (VAP) compared colistin monotherapy (19 patients) with colistin plus ampicillin-sulbactam at sulbactam dosing of 6 g per day (20 patients). Clinical response was defined as an improvement of symptoms for at least 48 h, a decrease in purulent bronchial secretions with vital sign improvement, or a consistent decrease in vasopressor use. Clinical response occurred in 15.8% of colistin patients compared with 70% of colistin plus ampicillin-sulbactam with no difference in mortality. Two large meta-analyses also found consistent positive outcomes with ampicillin-sulbactam combinations (62, 63). Liu and colleagues examined results from 18 studies in 1835 patients, of which less than 5% of ampicillin-sulbactam treated isolates were documented susceptible or intermediate, and found that ampicillin-sulbactam with sulbactam in doses of at least 6 g/day combined with another agent (levofloxacin or tigecycline) was the regimen most associated with clinical improvement and clinical cure but with no difference in mortality (62). Three agents were used to achieve sulbactam doses ≥ 6 g daily: (i) ampicillin-sulbactam dosed 3 g every 4 h (*n* = 1) or 6 g every 6 h (*n* = 2), (ii) sulbactam dosed 1.5 g every 4 h (*n* = 1) or 3 g every 8 h (*n* = 1), and (iii) cefoperazone-sulbactam, presumably dosed 4.5 g every 4 h (*n* = 1). Two studies utilized either ampicillin-sulbactam or cefoperazone-sulbactam dosed 6 g sulbactam daily without identifying the specific dosing regimen (64, 65). This same analysis of predominantly HAP/VAP, however, found that combinations including colistin with rifampicin, colistin with fosfomycin, or colistin with high-dose sulbactam were associated with improved microbiological eradication when compared with colistin monotherapy alone or high-dose sulbactam combined with an additional agent. An earlier review of 23 studies in 2,118 patients with MDR/XDR pneumonia identified that sulbactam-containing regimens were the most effective therapy in reducing all-cause mortality (59, 63). Available clinical evidence supports the inclusion of sulbactam at daily doses ≥ 6 g as part of a combination regimen targeting CRAB, given the demonstrated mortality benefit regardless of isolate susceptibility. Based on PK/PD data, higher doses of sulbactam (i.e., daily doses of 9 g) can be considered for intermediate isolates, although this still may not sufficiently achieve target attainment for resistant isolates (MIC ≥16 mcg/mL). Current global susceptibility data signal that sulbactam-durlobactam may be the more appropriate empiric sulbactam-containing option, given the likelihood of non-susceptibility to ampicillin-sulbactam among CRAB isolates.

### Tigecycline

Tigecycline is a 9-t-butylglycylamido derivative of minocycline (66). Tigecycline is able to overcome *tetM*-mediated tetracycline resistance through its ability to bind to ribosomal sites with greater affinity (67).

Recent data from 2016 to 2021 reported a global MIC90 of 2 mcg/mL and MIC50 of 1 mcg/mL for tigecycline among *A. baumannii* (68, 69); however, interpretation of these MICs is hindered by the lack of established breakpoints for *Acinetobacter* spp. by any breakpoint-setting organization. Two pharmacokinetic and pharmacodynamic studies have documented that higher doses are needed for MICs ≥ 2 mcg/mL (70, 71). Both studies utilizing pharmacokinetic data from critically ill patients found >90% probability of target attainment (AUC/MIC ≥4.5) for *Acinetobacter* spp. isolates with MICs ≤ 1 mcg/mL for the treatment of HAP using the standard tigecycline dose of 50 mg every 12 h. This probability fell to about 40% with a MIC of 2 mcg/mL (71). Increasing the tigecycline maintenance dose to 100 mg every 12 h was predicted to achieve >90% probability of target attainment for isolates with a MIC of 2 mcg/mL (72).

Several retrospective, clinical studies have further highlighted poor clinical outcomes for *A. baumannii* infections with regular dosing of tigecycline aligning with the need to use higher tigecycline doses and combination therapy (73–75). In a systematic review for *A. baumannii* pneumonia, high-dose tigecycline provided a more substantial survival benefit (71.4%) compared with standard dose tigecycline (28.6%) (63). Standard dose tigecycline was considered inferior to intravenous colistin in this analysis. Similarly, in a review of 10 studies with 593 patients across infections caused by various carbapenem-resistant gram-negative organisms (including 193 *A*. *baumannii i*solates), high-dose tigecycline provided mortality, clinical cure, and microbiologic eradication benefit without an increase in adverse effects (76). In addition, the mortality benefit favoring high-dose tigecycline was consistent across multiple subgroups including those with HAP/VAP, bacteremias, and carbapenem-resistant organisms. Until additional data become available and because of the pharmacokinetic challenges with this agent, only high-dose tigecycline in combination treatment regimens is recommended by IDSA and ESCMID for *A. baumannii* infections (22, 53).

Resistance to tigecycline in *A. baumannii* is complex and can be attributed to multiple different mechanisms. The most common mechanism reported is the overexpression of various efflux pump systems. This is often found in combination with altered permeability, altered antibiotic targets, and tigecycline-inactivating enzymes (77).

### Minocycline

Minocycline is a semisynthetic derivative of tetracycline. Among the tetracyclines, tigecycline and minocycline are the more potent tetracyclines compared with doxycycline for *A. baumannii,* especially for carbapenem-non-susceptible strains (78). Surveillance studies for *Acinetobacter* spp. isolates collected in the United States from 2014 to 2021 support the use of minocycline with *in vitro* susceptibility using a MIC ≤4 mcg/mL in 86% of all *A. baumannii* and in 66% of CRAB (79, 80). When combined with carbapenems, colistin, or rifampin, minocycline exhibits synergistic bactericidal activity against CRAB isolates (81). Minocycline achieves total serum levels of ~2.4 mcg/mL (range: 0.539–7.88 mcg/mL) after a 200 mg intravenous dose, which renders it a potentially more advantageous option based on pharmacokinetics for the treatment of *A. baumannii* severe infections, including bacteremias, compared with tigecycline (82). Limited clinical data, however, are available for minocycline for the treatment of *A. baumannii* infections, and the majority of the data are limited to combination therapy with other agents (83–85). In the largest retrospective study of 55 patients of which 85% were in the ICU and 71% were mechanically ventilated, minocycline was used in various combinations against CRAB infections with a clinical success of 73% (85). Only three patients received it as monotherapy. The most common double combinations included either intravenous colistin or ampicillin-sulbactam, and triple combinations included doripenem plus intravenous or inhaled colistin or ampicillin-sulbactam plus intravenous colistin. The infection-related mortality was 25%. The most common dosing strategy used (100 mg every 12 h), however, has been recently suggested to be too low based on PK/PD data.

An important consideration for incorporating minocycline into a combination regimen is the likelihood of adequate drug exposure based on the currently established breakpoints. These breakpoints have been called into question based on PK/PD evidence of suboptimal plasma exposure with *A. baumannii* MICs > 1 mcg/mL, even at high doses of 200 mg IV every 12 h (82). This is of broad relevance as a SENTRY surveillance study of 1,081 isolates from 2014 to 2018 showed that 68.7% of *A. baumannii-calcoaceticus* complex isolates had a MIC of ≤1 mcg/mL, which decreased to 40% in MDR isolates (80). Despite the concerning PK/PD data, *in vitro* modeling has demonstrated strong bactericidal effects when used at high doses (700 mg load, 350 mg every 12 h maintenance) in combination with polymyxin B and continuous infusion sulbactam for CRAB and in combination with continuous infusion meropenem +/− sulbactam for non-CRAB isolates (86). More data are needed to address how these differences in target attainment impact clinical outcomes. Minocycline possesses more favorable pharmacokinetics compared with tigecycline and should be preferred, although optimal dosing is yet to be established. Although breakpoints for minocycline continue to be re-evaluated, higher doses of 200 mg every 12 h should be considered in combination regimens for CRAB.

### Eravacycline

Eravacycline is a fully synthetic fluorocycline with structural modifications from tigecycline to the D-ring tetracycline core, resulting in improved *in vitro* activity against *A. baumannii* compared with tigecycline by a 2-fold to 8-fold MIC reduction (87–91). The implications of this difference are not well understood in the absence of CLSI breakpoints to interpret eravacycline susceptibility, as well as limited clinical data in CRAB infections, which include RCTs in complicated intra-abdominal infection and very few observational studies. A proposed epidemiological cutoff value for eravacycline for *A. baumannii* is 0.25 mcg/mL (92). For these reasons, the IDSA reserves the use of eravacycline in combination therapy in the absence of other active agents (53).

In a multicenter, retrospective observational study of patients on eravacycline regimens for *A. baumannii* infections conducted by Alosaimy and colleagues, 46 patients were treated for a median duration of 6.9 days (IQR: 5.1–11.1) with 84.4% receiving combination therapy (93). Eravacycline MIC values ranged from 0.125 to 1.50 mcg/mL; 69.5% were CRAB, and 58.3% were pulmonary infections. The 30-day mortality rates were 23.9% overall and 21.9% in patients with CRAB. Outcomes in pulmonary infections were also documented by Jackson and colleagues, in their retrospective study of 24 patients receiving eravacycline combination therapy (predominantly ampicillin/sulbactam and inhaled colistin) for CRAB VAP, showing clinical resolution in 71% of patients (94). It should be noted that MICs for eravacycline were not obtained. One of the few comparative studies of eravacycline to the best available therapy comes from Scott and colleagues who assessed the 30-day mortality rates in 93 patients with difficult-to-treat-resistant (DTR) *A. baumannii* pneumonia (95). The best available therapy included primarily beta-lactam monotherapy and beta-lactam plus intravenous or inhaled colistin. Eravacycline-based therapy resulted in higher 30-day mortality rates (33% vs 15%), longer durations of mechanical ventilation (10.5 v 6.5 days), and lower microbiologic cure (17% vs 59%). This study also included bacteremic patients, of which all four in the eravacycline arm died. A pooled analysis of patients with bacteremia caused by gram-negative pathogens in two phase 3 studies of complicated intra-abdominal infection, IGNITE1 and IGNITE4, found no difference in clinical cure (87.5% eravacycline vs 77% carbapenem comparator) or microbiologic response at 28 days (97.1% vs 97.2%) compared with carbapenem therapy. Of note, 100% of patients receiving eravacycline in the IGNITE 1 and 4 trials infected with *A. baumannii* (*n* = 8 IGNITE 1, *n* = 5 IGNITE 4) achieved favorable clinical response (96, 97). Existing clinical data suggest poor outcomes associated with eravacycline for CRAB, including bacteremia. In addition, the absence of breakpoints and PK/PD data to identify an optimal dose limits the use of this agent in clinical practice at this time.

Eravacycline maintains activity similar to tigecycline against resistance mechanisms mediated in earlier tetracyclines, namely ribosomal protection proteins and non-RND efflux pumps (98). As with tigecycline, RND family efflux pumps, the AdeABC system has been associated with reduced susceptibility or resistance. A significant correlation between increasing eravacycline MICs and overexpression of *adeB* efflux genes of *A. baumannii* has been identified, as well as *adeS* mutations (sensor responsible for expressing AdeABC pump) (89, 99).

### Cefiderocol

Cefiderocol, a broad-spectrum beta-lactam with a novel iron-carrier mechanism of action, was brought to market aimed at treating multidrug-resistant gram-negative infections including CRAB based on promising *in vitro* susceptibility data (100–102). Management for CRAB with cefiderocol monotherapy has remained controversial due to the findings of the CREDIBLE-CR trial. This multicenter, randomized, controlled trial of 118 patients with carbapenem-resistant gram-negative infections conducted a subgroup analysis of the 56 patients infected with CRAB and found higher all-cause mortality rates compared with best-available therapy (48.7% vs 17.6%) (51). Because of these findings, both the ESCMID and IDSA guidance recommend the use of cefiderocol only in CRAB infection refractory to other agents (22, 53). However, critiques of this analysis cite that additional comparisons accounting for baseline characteristics were not provided, wherein there were notable differences between the two treatment groups. This included more patients with ongoing shock and ICU patients at randomization in the cefiderocol group (19.6% vs 6.0% and 81.0% vs 47.0%, respectively) (103). Since the publication of CREDIBLE-CR, a growing body of literature has supported the use of cefiderocol in CRAB infections, including six observational studies evaluating mortality rates with cefiderocol for CRAB treatment. In a systematic review and meta-analysis conducted by Gatte and colleagues in 2023, including CREDIBLE-CR and four observational studies, mortality rates were not significantly reduced (104). However, when only including observational studies that adjusted for confounders, a significant reduction was observed (OR: 0.53; 95% CI: 0.39–0.71; I^2^ = 0.0%). These findings of mortality reduction have been echoed in other recent meta-analyses (105, 106). Although recommendations are to use cefiderocol in combination based on clinical outcomes and potential for resistance, which will be discussed below, some studies have demonstrated lower mortality rates of monotherapy compared with combination regimens as well (105). However, due to small sample sizes, retrospective study designs, and heterogeneity of included studies, further prospective investigations should be conducted to strengthen these authors’ findings.

The PROVE trial is an ongoing international, multicenter, retrospective chart review on the use of cefiderocol in carbapenem-resistant gram-negative infections. An interim analysis of 1,075 hospitalized patients showed high rates of ICU admission at 56.6% and *A. baumannii* as the second most common causative pathogen at 19.6% (107). They also reported clinical cure rates for all gram-negative organisms of 64% and response rates of 71.6% by the end of treatment, which had a median duration of 11 days. An earlier interim analysis of 244 patients identified 43 monomicrobial *A. baumannii* infections and reported clinical cure and clinical response rates of 64.6% and 74.4%, respectively (108). Whether the results of this real-world observational study alongside other recent observational data will impact international recommendations remains to be seen.

Most of the described cefiderocol resistance in CRAB has been classified as heteroresistance or a resistant subpopulation that appears susceptible and subsequently enriched under selective pressure (109, 110). This form of resistance is typically not detected by standard susceptibility testing and has been postulated to be another potential reason for the negative findings in the CREDIBLE-CR trial. However, newer studies have delivered conflicting evidence on this. A post hoc analysis of the CREDIBLE-CR study using population analysis profiling (PAP) to detect heteroresistance found that the highest clinical cure rates and lowest mortality rates came from the PAP-heteroresistant isolates (111). In contrast, Shields and colleagues found that among 27 CRAB isolates collected in the USA and Italy, rates of clinical failure were numerically higher in the cefiderocol-heteroresistant population (81% vs 55%). With these conflicting findings, the impact of heteroresistance is not well understood. It also remains uncertain how patient and environmental factors influence the impact of heteroresistance on clinical outcomes. In addition, concerns have been raised regarding the reliability of susceptibility testing of cefiderocol. Considering these limitations, the use of cefiderocol as part of combination therapy may be considered in the absence of other susceptible agents.

The development of cefiderocol resistance is mediated by several mechanisms, including reduced expression or mutations in siderophore receptor TBDR genes *piuA* and *pirA* as well as mutations in Acinetobacter-derived cephalosporinase PBP3 (112–115). From the standpoint of emerging resistance, cefiderocol may be a promising option due to its unique activity against metallo-beta-lactamase-producing CRAB, including IMP, VIM, and NDM, which continue to increase in prevalence in North America (116, 117). NDM-producing *A. baumannii* have been reported globally since 2005 (118–124). A 2023 multicenter study in Israel identified 54 of 857 CRAB isolates to express NDM (125). Despite known *in vitro* activity of cefiderocol against metallo-beta lactamases, NDM-1, -5, and -9 production has all been shown to partially contribute to decreased susceptibility (MICs of 1, 0.5, and 1 mcg/mL, respectively, in a recombinant lab strain). Mechanistically, co-expression of other previously described resistance elements together results in resistance (126).

### Sulbactam-durlobactam

Sulbactam-durlobactam is a novel combination of the beta-lactam (and beta-lactamase inhibitor) sulbactam and next-generation diazabicyclooctane (DBO) beta-lactamase inhibitor durlobactam approved by the FDA in 2023 specifically for hospital-acquired bacterial pneumonia and ventilator-associated bacterial pneumonia caused by susceptible isolates of *A. baumannii calcoaceticus* complex in adults (127). Durlobactam provides activity against commonly expressed beta-lactamases, notably for class D (OXA-23, 24/40) beta-lactamases, alongside class A and C (128). As with other DBO beta-lactamase inhibitors, durlobactam does not retain activity against metallo-beta-lactamases. The addition of durlobactam has been shown *in vitro* to restore the activity of sulbactam, lowering the MIC from 64 mcg/mL to 2 mcg/mL (30, 128–130). Durlobactam has also demonstrated intrinsic activity against some genera of Enterobacterales due to its ability to bind and inhibit PBP2. In the phase 3 multicenter randomized controlled trial, ATTACK, sulbactam-durlobactam in combination with background imipenem-cilastatin resulted in a 13.2% reduction in 28-day all-cause mortality compared with colistin therapy (131). The benefit of background carbapenem therapy for all CRAB infections is less clearly defined, although *in vitro* data have demonstrated a 1-fold to 2-fold MIC reduction in sulbactam-durlobactam when used in combination (132–134). Alongside imipenem in the ATTACK trial, published literature supports similar effects with meropenem (135–137). It has been theorized that the carbapenem component may serve to bind circulating OXA-carbapenemases, thereby preserving the activity of durlobactam and by extension sulbactam (138). Another proposed mechanism is that carbapenems may potentiate activity via expanded PBP binding. Carbapenems bind PBP2 alongside sulbactam binding PBP1 and 3 under the protection of durlobactam (132–134, 136). Sulbactam-durlobactam has not demonstrated appreciable synergy (strain-dependent synergy has been noted) with other commonly used agents for *A. baumannii* infections (139).

As a result of the significant mortality reduction seen in the ATTACK trial, sulbactam-durlobactam is now endorsed by IDSA as the preferred therapy for CRAB in combination with a carbapenem (53). For now, published real-world experience with sulbactam-durlobactam remains sparse, with all reports of use administered in combination with other agents and achieving clinical success. Combinations include sulbactam-durlobactam with cefiderocol, colistin, meropenem, and cefiderocol + minocycline triple therapy (115, 135, 138, 140). Although the utility of background therapy has been shown to demonstrate benefit in polymicrobial infection, sulbactam-durlobactam monotherapy remains an unanswered question for monomicrobial infection (141).

Resistance to sulbactam-durlobactam remains low at less than 1.7% of isolates collected between 2016 and 2021 (29). In another study assessing *in vitro* activity of isolates collected globally from 2016 and 2017, 2.3% of the isolates were resistant (30). Resistance mechanisms described include mutations in PBP3, the target of sulbactam, as well as previously described metallo-beta-lactamase production (130).

### Polymyxins

Polymyxin-based therapy, including colistin (polymyxin E) and polymyxin B, has historically been a staple component of regimens targeting multidrug-resistant gram-negative organisms, including CRAB (142). The basis of this practice was driven by the high *in vitro* susceptibility of CRAB isolates as well as demonstrated synergy with various other agents, including meropenem, imipenem, minocycline, and rifampin. However, more recent evaluations of polymyxin PK/PD data failed to determine a susceptibility breakpoint, and thus, only intermediate and resistant breakpoints of 4 mcg/mL and ≥8 mcg/mL are recognized (143). Despite almost all existing literature and *in vitro* synergy data being conducted with colistin, the IDSA guidance recommends the use of polymyxin B preferentially due to less toxicity (i.e., nephrotoxicity) and a more favorable pharmacokinetic profile. These features include more quickly achieving steady-state concentrations, not requiring prodrug activation, and less inter-patient variability in drug exposure (21, 53). High mortality rates with the use of colistin in clinical trials may be mechanistically explained by suboptimal exposures of colistin and evidence of regrowth and resistance development on monotherapy (144). The IDSA treatment guidance identifies polymyxin only as an additive agent in combination therapy, with sulbactam-based antimicrobials as the primary agent. Clinical data, including from randomized controlled trials, have also failed to identify the benefits of colistin combination therapy compared with colistin monotherapy. This includes two RCTs comparing colistin with colistin plus meropenem, as well as three RCTs comparing colistin with colistin plus rifampin (145–149). In one randomized 2018 study by Makris and colleagues comparing colistin monotherapy versus combination with high-dose ampicillin-sulbactam and inhaled colistin, 5-day clinical improvement was significantly higher with combination therapy (15.8% vs 70%, *P* = 0.01) (59). However, 28-day mortality was the same between groups with 63% in monotherapy and 50% in combination. An important consideration when interpreting these findings is the predominance of respiratory infections in this study, as pharmacokinetic concerns have been raised regarding adequate colistin delivery to the lungs (150, 151). Overall, meta-analyses evaluating clinical response in CRAB infections with or without colistin combination therapy have failed to find a substantial benefit of the combination compared with monotherapy (152). In focusing on polymyxin B-based combinations, one retrospective cohort study comparing monotherapy versus combination therapy in ICU patients with extensively drug-resistant CRAB or *Pseudomonas aeruginos*a infection demonstrated a mortality benefit in polymyxin B plus either carbapenem or beta-lactam therapy in lower respiratory tract infection (HR: 0.33, 95% CI: 0.17–0.66, *P* = 0.002) (153). These findings are echoed by other recent small observational studies (154). Although most of the clinical outcome data for polymyxins focus on colistin, polymyxin B is favored based on its pharmacokinetic profile for most infection types. Polymyxin use for the treatment of CRAB infections has fallen out of favor due to the availability of better-tolerated agents but may still be considered in the last-line cases. As previously discussed, clinical correlations with MIC results have limited the ability to define polymyxin susceptibility.

Polymyxin resistance in *A. baumannii* is primarily driven by modifications to lipid A biosynthesis, specifically via mutations in genes of two-component signaling proteins *pmrA* and *pmrB* (155). Experimental models have demonstrated that exposure to sublethal polymyxins quickly alters the lipid distribution in the cell wall to limit membrane destabilization, primarily through surface charge changes (156). Although surveillance studies have shown low rates of polymyxin resistance in CRAB (prior to breakpoint re-evaluation), evidence of resistant clonal expansion has been observed (157).

## SPECIFIC TREATMENT OPTIONS FOR CARBAPENEM-SUSCEPTIBLE *A. BAUMANNII*

None of the recently published guidelines address the treatment of carbapenem-susceptible *A. baumannii* infections and only focus on moderate-to-severe CRAB. In the case of highly susceptible isolates, clinicians are faced with the decision of whether to spare the first-line options in less severe infections, such as skin and soft tissue and urinary tract infections. In carbapenem-susceptible *A. baumannii* infections, ampicillin-sulbactam has been retrospectively compared with treatment with imipenem-cilastatin (158, 159). One study examined the two treatment options in 48 patients with *A. baumannii* bacteremia and found no differences in days of bacteremia, days to clinical resolution, success during or at the end of treatment, or ICU or total antibiotic-related length of stay (158). The other study reported 77 episodes of VAP, 14 treated with ampicillin-sulbactam compared with 63 treated with imipenem-cilastatin (159). Although all isolates in the imipenem-cilastatin group had carbapenem-susceptible isolates, only two of the 14 in the ampicillin-sulbactam group were carbapenem-susceptible. Patients treated with ampicillin-sulbactam were more likely to receive combination therapy with an aminoglycoside, but mortality, duration of mechanical ventilation, or length of stay in the intensive care unit or hospital were not different between the groups.

Routine susceptibility testing of *A. baumannii* includes additional beta-lactam agents including ceftazidime, cefepime, and piperacillin-tazobactam (160). Extended-spectrum cephalosporins that are stable against ADC hydrolysis should be considered when clinically indicated to slow the emergence of carbapenem resistance (161). Among these, very limited clinical data exist to support the comparative efficacy of ampicillin-sulbactam and carbapenem therapy. Chang and colleagues conducted a multicenter retrospective study in Taiwan comparing cefepime or cefpirome, another zwitterionic fourth-generation cephalosporin, with carbapenem therapy for monomicrobial *A. baumannii* bloodstream infection over a 6-year period. Among the subset of patients with *A. baumannii*, 30-day mortality rates were 50% (7/14) in the carbapenem group and 8.3% (1/12) in the cefepime-cefpirome group (*P* = 0.036) (162). Piperacillin-tazobactam presents an interesting option based on *in vivo* data showing intrinsic activity against *Acinetobacter* spp. isolates. However, when compared with sulbactam, both tazobactam and clavulanic acid were found to have inferior activity, and the current susceptibility testing methods using fixed concentrations may falsely predict higher activity or uninterpretable results (163). Imipenem or meropenem susceptibility should be confirmed prior to use, as discordant susceptibility results have been reported. The use of high-dose extended-infusion meropenem to maximize pharmacokinetic/pharmacodynamic parameters is an option (164). This dosing strategy has been studied compared with cefiderocol in the APEKS-NP clinical trial for nosocomial pneumonia. Cefiderocol was found to be non-inferior to high-dose extended-infusion meropenem overall, and in the subset of patients with *Acinetobacter* spp infections, clinical and microbiological outcomes were similar and not different based on meropenem MIC (165).

## CONSIDERATIONS BY THE SITE OF INFECTION

Each targeted agent has unique pharmacokinetic properties that permit advantages and disadvantages for use in certain sites of infection. This section will highlight each difference by drug class and the implications for treatment in urinary tract, bloodstream, and pulmonary infections and is further summarized in Table 1.

**TABLE 1:** Pharmacokinetic and clinical outcome highlights for new therapeutic options in the treatment of CRAB infections^*c*^

Agent	Pharmacokinetic considerations		Clinical outcomes by infection type			Benefit to combination?
	CSF	ELF	CNS	Pulmonary	Bloodstream	
Ampicillin-sulbactam	Up to 32% in meningitis and <1% in noninflamed meninges (166)	53% and 61% of serum levels for ampicillin and sulbactam, respectively (167)	In case series of 7 patients, one mortality. 1/3 with IT gentamicin (168) In case series of 12 patients, one mortality. 2 IT amikacin (169)	Demonstrated comparative benefit of sulbactam-based regimens on mortality reductions (63)	Successful use in case series: 40 consecutive patients, 32.5% bloodstream infections, overall mortality 17.5% (170) Similar outcomes in the study of 48 bacteremic patients compared with imipenem-cilastatin (158)	Meta-analyses identifying optimal combination regimens have found high-dose sulbactam monotherapy to be associated with mortality benefit (63) Majority of clinical data in combination
Tigecycline	Poor penetration, 11% (171) 5 mg intrathecal produces C_max_ of 37.8 mcg/mL and t_1/2_ of 2.73 hours (172)	200 mg loading dose, then 100 mg q12 resulted in median ELF C_max_ 0.42 mcg/mL and C_min_ 0.32 mcg/mL (72) ELF/plasma ratio of 152.9% (72)	Review of 50 cases, primarily with IT and IV polymyxin; seven deaths (173) 25% mortality rate in the study of four cases of CRAB meningitis (174)	Did not meet the primary endpoint in HAP RCT (175) FDA analysis revealed increased risk with HAP/VAP (176)	Pooled data from 13 RCTs demonstrate VAP with baseline bacteremia as increased risk of mortality (68)	*In vitro* synergy demonstrated with carbapenems, sulbactam, and polymyxin B (177, 178) In a meta-analysis of tigecycline use in patients with CRAB, no difference was observed between monotherapy and combination (179)
Minocycline	11-56% of plasma (180)	Mean lung tissue/plasma ratio of 2.92 mcg/g; mean sputum/serum ratio of 2.12 mcg/g (181) AUC_ELF_/AUC_serum_ of 2.5-2.8 in murine model (182)	One successful reported case: Sulbactam-durlobactam 6-week course with minocycline (4 weeks) and cefiderocol (6 weeks) (183)	Limited data, no comparative data: Published clinical review included 91 patients with infections of the respiratory tract and reported overall clinical success of 78% across all infection types (85)	Limited data, no comparative data: Largest retrospective study in MDR A. baumannii infections included nine bloodstream infections with 90% clinical success (most combination therapy) (85)	Majority of clinical data use minocycline in combination with other agents. High-dose minocycline, continuous-infusion sulbactam, and polymyxin B produced the most significant kill against the carbapenem-resistant *Acinetobacter baumannii* in an *in vitro* pharmacodynamic model (86)
Cefiderocol	4% CSF/plasma C_trough_ ratio, 68% AUC_CSF_/AUC_plasma_ (184, 185)	Low ELF concentrations ELF/total plasma 0.101 ELF/free plasma 0.239 (186)	Two successful reported cases: Cefiderocol 21-day course plus IVT gentamicin combo x 7d (185) Cefiderocol 21-day course plus sulbactam-durlobactam plus IVT polymyxin B (187)	No significant differences in mortality were observed compared with colistin when grouped by infection site, six observational studies OR 0.62 [0.33, 1.17] (106) CREDIBLE-CR: higher rates of all-cause mortality compared with the best available therapy in nosocomial pneumonia and BSI (51)	Lower mortality rates observed in meta-analyses when grouped by infection site, BSI OR 0.61 [0.41, 0.90] (106) CREDIBLE-CR: higher rates of all-cause mortality compared with best available therapy in nosocomial pneumonia and BSI (51)	Lower mortality rates with monotherapy have been described (105) *In vitro*, synergy described in combination with tigecycline and sulbactam (188)
Sulbactam-durlobactam (SUL-DUR)	10.3%/8.8%^*a*^ CSF/plasma C_max_ ratio (138)	86%/41.3% ELF/free plasma penetration ratios (189)	Three successful reported cases: Sulbactam-durlobactam 14-day course with meropenem (138) Sulbactam-durlobactam 6-week course with minocycline (4 weeks) and cefiderocol (6 weeks) (183) Sulbactam-durlobactam 21-day course plus cefiderocol plus IVT polymyxin B (187)	ATTACK trial: 41% HABP, 55% VABP in SUL-DUR arm. Significant reduction in mortality compared to the colistin group (19% vs 32%) (131)	Limited data: 2 patients in the ATTACK trial part A had BSI as the primary source of infection, both of whom expired^*b*^. 5/28 patients died at 28 days in Part B (131).	Absence of published data as monotherapy FDA approval regimen in combination with background carbapenem therapy. Carbapenems contribute to small MIC reductions in SUL-DUR. No appreciable synergy has been described (139)
Eravacycline	Poor penetration observed in rabbit PK studies (190)	6-fold greater concentrations in ELF than plasma in phase one study (191)	No reported cases	Case series of 24 patients with VAP, 17 (71%) achieved clinical resolution. All in combination (*n* = 23 with ampicillin-sulbactam, *n* = 7 with inhaled colistin) (94) In 93 patients with pneumonia, 30-day mortality was higher in the eravacycline arm compared to BAT (33% vs 15%). No difference was observed when excluding bacteremic patients. In all but one case, used in combination (95) 56% mortality rate amongst 18 patients treated for CRAB. 78% of patients were VAP. 28% received combination therapy (192) 46 patients, 58.3% pulmonary infections. 84.4% received combination. 23.9% mortality (93)	No comparative literature	Almost exclusively administered in combination as salvage therapy. No clinically appreciable synergy with CRAB active agents (193)

^*a*^ CSF sampling performed after reduction in meningeal inflammation (CSF, WBC, and protein normalization).

^*b*^ Authors note insufficient patients for a meaningful subgroup analysis.

^*c*^ AUC, area under the curve; BAT, best available therapy; BSI, bloodstream infection; CNS, central nervous system; CRAB, carbapenem-resistant *Acinetobacter baumannii*; CSF, cerebrospinal fluid; ELF, epithelial lining fluid; FDA, Food and Drug Administration; HABP, hospital-acquired bacterial pneumonia; HAP, hospital-acquired pneumonia; IT, intrathecal; IV, intravenous; IVT, intraventricular; MDR, multidrug-resistant; PK, pharmacokinetic; RCT, randomized controlled trial; VABP, ventilator-associated bacterial pneumonia; VAP, ventilator-associated pneumonia.

### Urinary tract

All beta-lactam targeted therapies, including ampicillin-sulbactam, cefiderocol, and sulbactam-durlobactam, sustain appreciable urinary concentrations, ranging from 75% excreted unchanged with ampicillin-sulbactam, 75%–85%/78% with sulbactam-durlobactam, and 90.6% with cefiderocol (194–196).

Tetracyclines present the greatest variability in urinary concentrations, ranging from 5% to 12% excreted unchanged with minocycline to 33% and 34% with tigecycline and eravacycline, respectively (8, 197, 198). Despite modest concentrations, minocycline maintains an FDA indication for the management of UTI, and tigecycline has several reports with clinical cure rates as high as 77.4% (199). Despite slightly lower urinary excretion of eravacycline (34% unchanged) compared with doxycycline (35%–60% unchanged), randomized controlled data have failed to meet noninferiority in complicated UTI (198, 200, 201). Eravacycline failed to meet noninferiority in the IGNITE3 trial for complicated UTI.

Colistin is the polymyxin of choice for UTI due to high concentrations in the urine as a result of renal clearance pathways (202). Polymyxin B is primarily cleared nonrenally with only about 4% recovered in urine (203). Recommendations for colistin over polymyxin B in UTI are endorsed by international consensus guidelines (202).

### Bloodstream

Concerns for adequate intravascular drug exposures center primarily around the tetracycline class due to their very high volume of distribution. Mean tigecycline C_max_ after 1 h of infusion of 100 mg is reported around 1.94 mcg/mL, declining to 0.11 mcg/mL and 0.07 mcg/mL at 12 and 24 h. After multiple doses of tigecycline 50 mg, steady-state C_max_ was reported as 0.62–0.72 mcg/mL, well below the proposed clinically acceptable breakpoint of 1 mcg/mL (204, 205). Clinical studies have corroborated the concerns around low blood levels with documented increased mortality associated with tigecycline-based therapy for breakthrough *Acinetobacter* spp. bacteremia and the occurrence of breakthrough CRAB bacteremia while on tigecycline therapy (206, 207). Minocycline has a lower volume of distribution of 1.3 L/kg compared with 7–9 L/kg for tigecycline, and minocycline serum levels offer more promise compared with tigecycline. Following a single minocycline 200 mg dose, serum concentrations of minocycline ranged from 2.52 to 6.63 mcg/mL (average 4.18 mcg/mL) at the end of infusion and 0.82 to 2.64 mcg/mL (average 1.38 mcg/mL) after 12 h (208). Clinical outcomes supporting minocycline for the treatment of CRAB bacteremia are limited and primarily describe patients treated with minocycline combination regimens, secondary bacteremias from a respiratory source, and treatment with the lower minocycline dose of 100 mg twice daily (84). As with other tetracyclines, the excellent tissue penetration of eravacycline raises concerns for the treatment of intravascular infections. Eravacycline C_max_ concentrations are low and reported as 1,825–2,125 ng/mL after 1 mg/kg dosing. Eravacycline clinical data for the treatment of bacteremia are even more limited, often including secondary bacteremia from intra-abdominal infections not specific to *Acinetobacter* spp., and similarly describe use in combination with additional agents when used for CRAB (95, 96, 209, 210). Until additional data become available, tigecycline, minocycline, and eravacycline cannot be recommended as monotherapy for patients with bacteremia caused by *Acinetobacter* spp., but minocycline more so than eravacycline may be considered as part of a combination regimen.

### Pulmonary

Sulbactam-durlobactam and ampicillin-sulbactam have demonstrated efficacy in pulmonary infections. A population PK model for sulbactam-durlobactam using pooled phase 1, 2, and 3 data (eight total studies) including a total of 432 subjects was recently published. In epithelial lining fluid (ELF) sub-models, penetration relative to free-drug plasma was 41.3% for durlobactam and 86.0% for sulbactam (189). Of the targeted beta-lactam agents, cefiderocol stands out as raising concerns for adequate exposure in pulmonary infection and as a treatment option for pneumonia. Suboptimal penetration of cefiderocol in ELF has been demonstrated and has associated clinical implications, including meta-analysis findings of lower mortality rates with cefiderocol in bloodstream infections but not VAP (106, 186, 211). In the APEKS-NP study, a randomized controlled trial comparing cefiderocol to high-dose, extended-infusion meropenem (2 g every 8 h infused over 3 h) in 298 patients with gram-negative nosocomial pneumonia, cefiderocol demonstrated noninferiority in terms of all-cause mortality at day 14 (165). *A. baumannii,* which comprised 16% of baseline gram-negative pathogens, reported similar all-cause mortality (19% cefiderocol vs. 22% meropenem, treatment difference –3%, 95% CI –24.8 to 18.8). An important consideration is that this study included meropenem-non-susceptible isolates (24% in the cefiderocol group and 20% in the meropenem group), suggesting similar outcomes of cefiderocol compared with an agent that was inactive.

Of the tetracycline derivatives, minocycline has the most promise as an agent for pneumonias. Although the largest outcome study was small (*N* = 55), retrospective, utilized a relatively low dose of minocycline (100 mg every 12 h), and most commonly used minocycline in combination, clinical success was >70% in infections caused by multidrug-resistant *A. baumannii* (85). The most common site of infection was pneumonia with or without bacteremia in 65%. Concentrations of minocycline in lung tissue and ELF consistently exceed serum concentrations by at least 2-fold (181, 182). In a neutropenic murine pneumonia model, minocycline AUC_ELF,0-24_/AUC _serum,0-24_ ratio ranged from 2.5 to 2.8 (182). Although the pharmacokinetics are favorable for the treatment of pneumonias, optimal dosing remains to be established, and clinical data are limited to combination therapy with multiple combinations, such as combinations with carbapenems, sulbactam, and polymyxins (82).

As discussed earlier, the PK properties of tigecycline limit its utility for pneumonias and bacteremias, especially as monotherapy. Tigecycline has a large volume of distribution into tissues, which results in low serum concentrations (204). Data from 13 phase 3 and 4 studies demonstrated that tigecycline all-cause mortality was higher compared with comparator groups, most notably in patients with VAP and baseline bacteremia (68). Another study based on serum and ELF concentrations in patients receiving tigecycline 100 mg every 12 h found even lower probabilities of target attainment such that higher tigecycline doses could only achieve the optimal target at MICs ≤ 0.5 mcg/mL (72). In this same study, high-dose tigecycline resulted in median ELF C_max_ of 0.42 mcg/mL and C_min_ of 0.32, correlating to a median ELF to plasma ratio of 152.9%.

Eravacycline, although also exhibiting similarly suboptimal serum levels, showed more promise in pulmonary infections based on early PK studies, including a phase 1 PK study with ELF concentrations 6-fold higher than serum levels (191). These findings, however, have been difficult to translate clinically. In the retrospective study conducted by Scott and colleagues comparing eravacycline to the best available therapy for CRAB pneumonia, eravacycline was associated with increased mortality (however, when excluding bacteremic patients, showed no difference) (95). Non-comparative case series may also signal benefits of including eravacycline as part of a combination regimen for pneumonia (93, 94, 191). Although PK characteristics and reported cases support eravacycline use over tigecycline in pulmonary infections, further research is needed to confirm its benefit compared with other combinations.

PK/PD studies of colistin have demonstrated a low likelihood of achieving pulmonary concentrations at the previous breakpoint of 2 mcg/mL (212). International guidelines state that unless the MIC of the bacterial strain is well below the breakpoint, colistin is likely associated with suboptimal exposure in the lung (202). Similar effects have been demonstrated with polymyxin B, although less overall PK literature exists (213). Inhaled colistin or polymyxin B in combination with systemic therapy has not demonstrated benefits in clinical studies (214, 215). However, newer trials seek to adjust for potential design flaws, including suboptimal inhaled doses and uniform nebulization techniques and ventilator settings for drug delivery (216).

### Central nervous system

For carbapenem-susceptible isolates*, in vitro* and pharmacodynamic data support the use of meropenem dosed 2 g every 8 h administered by prolonged infusion. This aligns with the IDSA recommendations for empiric therapy for post-neurosurgical meningitis, which includes vancomycin plus either cefepime, ceftazidime, or meropenem (217). Pharmacodynamic assessment of both cephalosporins reveals that at recommended doses, both are likely to achieve necessary targets against less than 10% of *Acinetobacter* spp. isolates (218). Additionally, imipenem has been successfully used to treat meningitis; however, clinically significant seizures have been reported with use during treatment (168, 218). Lodise and colleagues conducted population PK modeling, which showed dosing meropenem at 2 g every 8 h infused over 3–4 h resulted in a probability of achieving 100% of time above MIC > 80% for MICs of 0.25 mcg/mL and <70% for MICs ≥ 0.5 mcg/mL (219). One gram of sulbactam has been shown to achieve CSF concentrations up to 32% of serum in patients with meningitis and less than 1% in those without meningitis (166). Concentrations of sulbactam in the central nervous system of up to 12 mcg/mL after single 1 g sulbactam doses or multiple doses administered every 6 h have been reported, suggesting potential benefit in cases of meningitis (220). A case series has documented the successful use of ampicillin-sulbactam with susceptible isolates at daily doses of sulbactam as low as 4 grams. Jiminez-Mejias and colleagues reported a series of patients receiving sulbactam dosed 1 g every 6 h, resulting in six of seven patients achieving clinical cure (168). It should be noted that over a third of reported patients also received intraventricular aminoglycosides. Cefiderocol has considerable variability reported for CSF penetration, ranging from 4% to 68% (184, 185). Two separate case reports addressing the pharmacokinetics of cefiderocol in CSF observed minimal fluctuations in CSF concentrations compared with serum (185, 187). Successful management of CRAB meningitis/ventriculitis with cefiderocol combination therapy has been reported (138, 185, 221).

Sulbactam-durlobactam may also be effective in the treatment of CNS infections. It was successfully used in the treatment of a case of CRAB meningitis (138). The patient was initially treated with high-dose ampicillin-sulbactam in combination with cefiderocol, but CSF cultures remained positive for 13 days. CSF clearance was only achieved following the initiation of sulbactam-durlobactam in combination with meropenem. At 3 h post-infusion, sulbactam and durlobactam CSF concentrations reached 1.36 µg/mL and 1.62 µg/mL, respectively. The percent concentration of serum reached was approximately 10.3% and 8.8%, respectively. The authors concluded that, based on the structural similarity with avibactam, in which CSF penetration ranges from 10% to 40% with successful clinical use in meningitis, durlobactam may predictably behave similarly. In another case report of meningitis and epidural empyema due to OXA-producing *A. baumannii*, the clinical cure was achieved after adding sulbactam-durlobactam in combination with cefiderocol and minocycline (183).

Among the tetracycline class, minocycline is the most lipophilic, permitting improved penetration into the CSF, at 11%–56% of plasma concentrations (180). Clinical data for the efficacy of minocycline in CNS infections remain extremely limited, with one reported successful case of use in combination with sulbactam-durlobactam and cefiderocol in a post-neurosurgical infection (183). Tigecycline penetration into the CNS for the treatment of meningitis/ventriculitis is known to be poor (11%) (171). Several case reports have documented the use of intrathecal or intraventricular use of tigecycline for *A. baumannii* CNS infections (173). In these cases, tigecycline was administered in doses of 2–10 mg administered either once or twice daily without any safety signals. Concomitant intravenous tigecycline was often continued, and additional antibiotics administered intravenously or directly instilled into the CNS were commonly used. Intrathecal or intraventricular tigecycline remains a treatment option for *A. baumannii* CNS infections in combination with other agents when resistance limits treatment options. Eravacycline displays a very high volume of distribution at 321 L/kg (198). Despite this, PK studies in rabbits support negligible penetration into the CSF.

Although polymyxins have limited penetration into the cerebrospinal fluid (CSF), intraventricular and intrathecal polymyxins have documented clinical success in multidrug-resistant *A. baumannii* meningitis and are endorsed by the IDSA for the management of MDR gram-negative meningitis (53). Prior studies suggest that in cases of multidrug-resistant nosocomial meningitis, intrathecal introduction of antibiotics improves survival outcomes (222). In a case series by Falagas and colleagues of 64 cases of gram-negative meningitis, 10 of the 11 cases caused by *Acinetobacter* spp. achieved cure (223). The most common toxicity was meningeal irritation, which occurred in 12 of 60 patients. In another retrospective study of 51 post-neurosurgical cases, all eight patients treated with intravenous and intrathecal colistin survived (224). In a more recent case series of six patients with XDR *A. baumannii* meningitis and ventriculitis, all patients sterilized CSF cultures with systemic and adjunctive intraventricular colistin (225). The recommended dosage is intraventricular polymyxin B 5 mg daily and intraventricular colistin 10 mg daily.

Alongside polymyxins, intraventricular aminoglycosides have also been employed to mitigate low CSF penetration of systemic antibiotics. Kim and colleagues reviewed case reports of intraventricular aminoglycosides for the treatment of *Acinetobacter* spp. meningitis, with 79% achieving clinical cure (218). This strategy is typically paired with systemic antibiotic administration of carbapenems or polymyxins. The IDSA guidelines recommend a daily intraventricular gentamicin dose of 4–8 mg. Rahal and colleagues demonstrated that at a dose of 4 mg intrathecally, CSF concentrations ranged from 19 to 46 mcg/mL (226). Based on the pharmacokinetic principle that the efficacy of aminoglycosides is driven by a C_max_ to MIC ratio of greater than 10, this dosing likely will reach optimal targets for most isolates as the current CLSI breakpoint is 4 mcg/mL. Toxicity data do not support the use of dosing beyond 10 mg, at which seizures and transient hearing loss have been seen. Guidelines also support the use of amikacin, which has higher documented *in vitro* activity against *Acinetobacter* spp. than gentamicin. However, intraventricular dosing studies are limited. Peaks ranging from 150 mcg/mL after 8 mg and >100 mcg/mL after 5 mg have been reported (227, 228). These were findings from pediatric patients, which further complicates the conclusion in adults; however, at the current breakpoint of 16 mcg/mL, the data are promising for achieving pharmacodynamic targets.

### Final summary

The therapeutic challenges of treating *Acinetobacter* spp. infections focus on minimizing the development of resistance and achieving meaningful reductions in mortality. Each of the various agents reviewed has strengths and weaknesses when being considered part of a combination regimen, which remains the recommended approach to managing severe infections. Clinical outcomes and attainment of pharmacokinetic targets are heterogeneous across infection types for many agents. As discussed here and in the 2024 IDSA update, sulbactam-durlobactam, in combination with background carbapenem therapy, remains the combination with the greatest reduction in mortality for pulmonary infections and has promising outcomes in bloodstream infections with CRAB. Sulbactam-based combination therapy remains an ideal part of targeted strategies and has been associated with reduced mortality. Certain agents have been highlighted in the literature for suboptimal outcomes, primarily pulmonary infections treated with cefiderocol, tigecycline, and eravacycline. Studies including non-pulmonary infections, specifically bacteremia and CNS infections, are overall limited to case series and subgroup analyses. All agents discussed, except eravacycline, have been incorporated into regimens targeting CNS infections. The majority of that literature describes the use of intraventricular antibiotics. Important areas for further research include breakpoint evaluations for eravacycline and minocycline as well as subclinical resistance in cefiderocol.

## References

[1] Dijkshoorn L, van der Toorn J. 1992. Acinetobacter species: which do we mean? Clin Infect Dis 15:748–749. doi:10.1093/clind/15.4.748 1420704

[2] Nemec A, Krizova L, Maixnerova M, Sedo O, Brisse S, Higgins PG. 2015. Acinetobacter seifertii sp. nov., a member of the Acinetobacter calcoaceticus-Acinetobacter baumannii complex isolated from human clinical specimens. Int J Syst Evol Microbiol 65:934–942. doi:10.1099/ijs.0.000043 25563912

[3] Cosgaya C, Marí-Almirall M, Van Assche A, Fernández-Orth D, Mosqueda N, Telli M, Huys G, Higgins PG, Seifert H, Lievens B, Roca I, Vila J. 2016. Acinetobacter dijkshoorniae sp. nov., a member of the Acinetobacter calcoaceticus-Acinetobacter baumannii complex mainly recovered from clinical samples in different countries. Int J Syst Evol Microbiol 66:4105–4111. doi:10.1099/ijsem.0.001318 27432448

[4] Chusri S, Chongsuvivatwong V, Rivera JI, Silpapojakul K, Singkhamanan K, McNeil E, Doi Y. 2014. Clinical outcomes of hospital-acquired infection with Acinetobacter nosocomialis and Acinetobacter pittii. Antimicrob Agents Chemother 58:4172–4179. doi:10.1128/AAC.02992-14 24820079 PMC4068534

[5] Tacconelli E, Carrara E, Savoldi A, Harbarth S, Mendelson M, Monnet DL, Pulcini C, Kahlmeter G, Kluytmans J, Carmeli Y, Ouellette M, Outterson K, Patel J, Cavaleri M, Cox EM, Houchens CR, Grayson ML, Hansen P, Singh N, Theuretzbacher U, Magrini N, WHO Pathogens Priority List Working Group. 2018. Discovery, research, and development of new antibiotics: the WHO priority list of antibiotic-resistant bacteria and tuberculosis. Lancet Infect Dis 18:318–327. doi:10.1016/S1473-3099(17)30753-3 29276051

[6] WHO. 2024. WHO Bacterial Priority Pathogens List, 2024: bacterial pathogens of public health importance to guide research, development and strategies to prevent and control antimicrobial resistance. World Health Organization, Geneva.

[7] CDC. 2019. Antibiotic resistance threats in the United States*.* US Department of Health and Human Services, Atlanta, GA.

[8] CDC. 2010. Tygacil [package insert]. Wyeth Pharmaceuticals Inc, Philadelphia, PA.

[9] Fournier PE, Richet H. 2006. The epidemiology and control of Acinetobacter baumannii in health care facilities. Clin Infect Dis 42:692–699. doi:10.1086/500202 16447117

[10] Playford EG, Craig JC, Iredell JR. 2007. Carbapenem-resistant Acinetobacter baumannii in intensive care unit patients: risk factors for acquisition, infection and their consequences. J Hosp Infect 65:204–211. doi:10.1016/j.jhin.2006.11.010 17254667

[11] Maragakis LL, Perl TM. 2008. Acinetobacter baumannii: epidemiology, antimicrobial resistance, and treatment options. Clin Infect Dis 46:1254–1263. doi:10.1086/529198 18444865

[12] CDC. 2021. HAI pathogens and antimicrobial resistance report 2018-2021, on US Department of Health and Human Services. https://www.cdc.gov/nhsn/datastat/ar-pathogens.html.

[13] Russo A, Giuliano S, Ceccarelli G, Alessandri F, Giordano A, Brunetti G, Venditti M. 2018. Comparison of septic shock due to multidrug-resistant Acinetobacter baumannii or Klebsiella pneumoniae carbapenemase-producing K. pneumoniae in intensive care unit patients. Antimicrob Agents Chemother 62:e02562-17. doi:10.1128/AAC.02562-17 29555630 PMC5971575

[14] Shorr AF, Zilberberg MD, Micek ST, Kollef MH. 2014. Predictors of hospital mortality among septic ICU patients with Acinetobacter spp.bacteremia: a cohort study. BMC Infect Dis 14:572. doi:10.1186/s12879-014-0572-6 25358621 PMC4216657

[15] Tokur ME, Korkmaz P, Alkan S, Yıldız E, Arık Ö, Renders DP, Balcı C. 2022. Mortality predictors on the day of healthcare-associated Acinetobacter baumanni bacteremia in intensive care unit. J Infect Dev Ctries 16:1473–1481. doi:10.3855/jidc.16902 36223624

[16] Falcone M, Tiseo G, Carbonara S, Marino A, Di Caprio G, Carretta A, Mularoni A, Mariani MF, Maraolo AE, Scotto R, et al.. 2023. Mortality attributable to bloodstream infections caused by different carbapenem-resistant gram-negative bacilli: results from a nationwide study in Italy (ALARICO Network). Clin Infect Dis 76:2059–2069. doi:10.1093/cid/ciad100 36801828

[17] Lemos EV, de la Hoz FP, Einarson TR, McGhan WF, Quevedo E, Castañeda C, Kawai K. 2014. Carbapenem resistance and mortality in patients with Acinetobacter baumannii infection: systematic review and meta-analysis. Clin Microbiol Infect 20:416–423. doi:10.1111/1469-0691.12363 24131374

[18] Pogue JM, Zhou Y, Kanakamedala H, Cai B. 2022. Burden of illness in carbapenem-resistant Acinetobacter baumannii infections in US hospitals between 2014 and 2019. BMC Infect Dis 22:36. doi:10.1186/s12879-021-07024-4 34991499 PMC8740340

[19] Appaneal HJ, Lopes VV, LaPlante KL, Caffrey AR. 2022. Treatment, clinical outcomes, and predictors of mortality among a national cohort of admitted patients with Acinetobacter baumannii infection. Antimicrob Agents Chemother 66:e0197521. doi:10.1128/AAC.01975-21 35007134 PMC8923192

[20] Vincent JL, Rello J, Marshall J, Silva E, Anzueto A, Martin CD, Moreno R, Lipman J, Gomersall C, Sakr Y, Reinhart K, EIGo I. 2009. International study of the prevalence and outcomes of infection in intensive care units. JAMA 302:2323–2329. doi:10.1001/jama.2009.1754 19952319

[21] Shields RK, Paterson DL, Tamma PD. 2023. Navigating available treatment options for carbapenem-resistant Acinetobacter baumannii-calcoaceticus complex infections. Clin Infect Dis 76:S179–S193. doi:10.1093/cid/ciad094 37125467 PMC10150276

[22] Paul M, Carrara E, Retamar P, Tängdén T, Bitterman R, Bonomo RA, de Waele J, Daikos GL, Akova M, Harbarth S, Pulcini C, Garnacho-Montero J, Seme K, Tumbarello M, Lindemann PC, Gandra S, Yu Y, Bassetti M, Mouton JW, Tacconelli E, Rodríguez-Baño J. 2022. European Society of Clinical Microbiology and Infectious Diseases (ESCMID) guidelines for the treatment of infections caused by multidrug-resistant Gram-negative bacilli (endorsed by European society of intensive care medicine). Clin Microbiol Infect 28:521–547. doi:10.1016/j.cmi.2021.11.025 34923128

[23] Vivo A, Fitzpatrick MA, Suda KJ, Jones MM, Perencevich EN, Rubin MA, Ramanathan S, Wilson GM, Evans ME, Evans CT. 2022. Epidemiology and outcomes associated with carbapenem-resistant Acinetobacter baumannii and carbapenem-resistant Pseudomonas aeruginosa: a retrospective cohort study. BMC Infect Dis 22:491. doi:10.1186/s12879-022-07436-w 35610601 PMC9128216

[24] Seifert H, Baginski R, Schulze A, Pulverer G. 1993. Antimicrobial susceptibility of Acinetobacter species. Antimicrob Agents Chemother 37:750–753. doi:10.1128/AAC.37.4.750 8494371 PMC187751

[25] Vila J, Marcos A, Marco F, Abdalla S, Vergara Y, Reig R, Gomez-Lus R, Jimenez de Anta T. 1993. In vitro antimicrobial production of beta-lactamases, aminoglycoside-modifying enzymes, and chloramphenicol acetyltransferase by and susceptibility of clinical isolates of Acinetobacter baumannii. Antimicrob Agents Chemother 37:138–141. doi:10.1128/AAC.37.1.138 8431011 PMC187622

[26] Xie R, Zhang XD, Zhao Q, Peng B, Zheng J. 2018. Analysis of global prevalence of antibiotic resistance in Acinetobacter baumannii infections disclosed a faster increase in OECD countries. Emerg Microbes Infect 7:31. doi:10.1038/s41426-018-0038-9 29535298 PMC5849731

[27] Seifert H, Blondeau J, Lucaßen K, Utt EA. 2022. Global update on the in vitro activity of tigecycline and comparators against isolates of Acinetobacter baumannii and rates of resistant phenotypes (2016-2018). J Glob Antimicrob Resist 31:82–89. doi:10.1016/j.jgar.2022.08.002 35948242

[28] Bulens SN, Campbell D, McKay SL, Vlachos N, Burgin A, Burroughs M, Padila J, Grass JE, Jacob JT, Smith G, et al.. 2024. Carbapenem-resistant Acinetobacter baumannii complex in the United States-An epidemiological and molecular description of isolates collected through the Emerging Infections Program, 2019. Am J Infect Control 52:1035–1042. doi:10.1016/j.ajic.2024.04.184 38692307 PMC11979794

[29] Karlowsky JA, Hackel MA, McLeod SM, Miller AA. 2022. In vitro activity of sulbactam-durlobactam against global isolates of Acinetobacter baumannii-calcoaceticus complex collected from 2016 to 2021. Antimicrob Agents Chemother 66:e0078122. doi:10.1128/aac.00781-22 36005804 PMC9487466

[30] McLeod SM, Moussa SH, Hackel MA, Miller AA. 2020. In vitro activity of sulbactam-durlobactam against Acinetobacter baumannii-calcoaceticus complex isolates collected globally in 2016 and 2017. Antimicrob Agents Chemother 64:e02534-19. doi:10.1128/AAC.02534-19 31988095 PMC7179289

[31] Bonomo RA, Szabo D. 2006. Mechanisms of multidrug resistance in Acinetobacter species and Pseudomonas aeruginosa. Clin Infect Dis 43 Suppl 2:S49–56. doi:10.1086/504477 16894515

[32] Jacoby GA. 2009. AmpC beta-lactamases. Clin Microbiol Rev 22:161–182. doi:10.1128/CMR.00036-08 19136439 PMC2620637

[33] Wu HJ, Xiao ZG, Lv XJ, Huang HT, Liao C, Hui CY, Xu Y, Li HF. 2023. Drug‑resistant Acinetobacter baumannii: from molecular mechanisms to potential therapeutics (Review). Exp Ther Med 25:209. doi:10.3892/etm.2023.11908 37090073 PMC10119666

[34] Joshi SG, Litake GM, Ghole VS, Niphadkar KB. 2003. Plasmid-borne extended-spectrum beta-lactamase in a clinical isolate of Acinetobacter baumannii. J Med Microbiol 52:1125–1127. doi:10.1099/0022-1317-52-12-1125 14614072

[35] Vahaboglu H, Oztürk R, Aygün G, Coşkunkan F, Yaman A, Kaygusuz A, Leblebicioglu H, Balik I, Aydin K, Otkun M. 1997. Widespread detection of PER-1-type extended-spectrum beta-lactamases among nosocomial Acinetobacter and Pseudomonas aeruginosa isolates in Turkey: a nationwide multicenter study. Antimicrob Agents Chemother 41:2265–2269. doi:10.1128/AAC.41.10.2265 9333059 PMC164104

[36] Nagano N, Nagano Y, Cordevant C, Shibata N, Arakawa Y. 2004. Nosocomial transmission of CTX-M-2 beta-lactamase-producing Acinetobacter baumannii in a neurosurgery ward. J Clin Microbiol 42:3978–3984. doi:10.1128/JCM.42.9.3978-3984.2004 15364979 PMC516360

[37] Huang Z, Mao P, Chen Y, Wu L, Wu J. 2004. Study on the molecular epidemiology of SHV type beta-lactamase-encoding genes of multiple-drug-resistant Acinetobacter baumannii. Zhonghua Liu Xing Bing Xue Za Zhi 25:425–427. 15231171

[38] Ramirez MS, Bonomo RA, Tolmasky ME. 2020. Carbapenemases: transforming Acinetobacter baumannii into a yet more dangerous menace. Biomolecules 10:720. doi:10.3390/biom10050720 32384624 PMC7277208

[39] Castanheira M, Mendes RE, Gales AC. 2023. Global epidemiology and mechanisms of resistance of Acinetobacter baumannii-calcoaceticus complex. Clin Infect Dis 76:S166–S178. doi:10.1093/cid/ciad109 37125466 PMC10150277

[40] Iovleva A, Mustapha MM, Griffith MP, Komarow L, Luterbach C, Evans DR, Cober E, Richter SS, Rydell K, Arias CA, Jacob JT, Salata RA, Satlin MJ, Wong D, Bonomo RA, van Duin D, Cooper VS, Van Tyne D, Doi Y. 2022. Carbapenem-resistant Acinetobacter baumannii in U.S. hospitals: diversification of circulating lineages and antimicrobial resistance. MBio 13:e0275921. doi:10.1128/mbio.02759-21 35311529 PMC9040734

[41] Poirel L, Naas T, Nordmann P. 2010. Diversity, epidemiology, and genetics of class D beta-lactamases. Antimicrob Agents Chemother 54:24–38. doi:10.1128/AAC.01512-08 19721065 PMC2798486

[42] Evans BA, Hamouda A, Amyes SGB. 2013. The rise of carbapenem-resistant Acinetobacter baumannii. Curr Pharm Des 19:223–238. 22894617

[43] Tian J, Zhang G, Ju Y, Tang N, Li J, Jia R, Feng J. 2018. Five novel carbapenem-hydrolysing OXA-type β-lactamase groups are intrinsic in Acinetobacter spp. J Antimicrob Chemother 73:3279–3284. doi:10.1093/jac/dky359 30247543

[44] Müller C, Reuter S, Wille J, Xanthopoulou K, Stefanik D, Grundmann H, Higgins PG, Seifert H. 2023. A global view on carbapenem-resistant Acinetobacter baumannii. MBio 14:e0226023. doi:10.1128/mbio.02260-23 37882512 PMC10746149

[45] Quale J, Bratu S, Landman D, Heddurshetti R. 2003. Molecular epidemiology and mechanisms of carbapenem resistance in Acinetobacter baumannii endemic in New York City. Clin Infect Dis 37:214–220. doi:10.1086/375821 12856214

[46] Clark RB. 1996. Imipenem resistance among Acinetobacter baumannii: association with reduced expression of a 33-36 kDa outer membrane protein. J Antimicrob Chemother 38:245–251. doi:10.1093/jac/38.2.245 8877538

[47] Rizk MA, Abou El-Khier NT. 2019. Aminoglycoside resistance genes in Acinetobacter baumannii clinical isolates. Clin Lab 65. doi:10.7754/Clin.Lab.2019.190103 31307186

[48] Jouybari MA, Ahanjan M, Mirzaei B, Goli HR. 2021. Role of aminoglycoside-modifying enzymes and 16S rRNA methylase (ArmA) in resistance of Acinetobacter baumannii clinical isolates against aminoglycosides. Rev Soc Bras Med Trop 54:e05992020. doi:10.1590/0037-8682-0599-2020 33533819 PMC7849326

[49] Kornelsen V, Kumar A. 2021. Update on multidrug resistance efflux pumps in Acinetobacter spp. Antimicrob Agents Chemother 65:e0051421. doi:10.1128/AAC.00514-21 33903107 PMC8218648

[50] Novović K, Jovčić B. 2023. Colistin resistance in Acinetobacter baumannii: molecular mechanisms and epidemiology. Antibiotics (Basel) 12:516. doi:10.3390/antibiotics12030516 36978383 PMC10044110

[51] Bassetti M, Echols R, Matsunaga Y, Ariyasu M, Doi Y, Ferrer R, Lodise TP, Naas T, Niki Y, Paterson DL, Portsmouth S, Torre-Cisneros J, Toyoizumi K, Wunderink RG, Nagata TD. 2021. Efficacy and safety of cefiderocol or best available therapy for the treatment of serious infections caused by carbapenem-resistant Gram-negative bacteria (CREDIBLE-CR): a randomised, open-label, multicentre, pathogen-focused, descriptive, phase 3 trial. Lancet Infect Dis 21:226–240. doi:10.1016/S1473-3099(20)30796-9 33058795

[52] Tamma PD, Aitken SL, Bonomo RA, Mathers AJ, van Duin D, Clancy CJ. 2023. Infectious Diseases Society of America 2023 guidance on the treatment of antimicrobial resistant Gram-negative infections. Clin Infect Dis:ciad428. doi:10.1093/cid/ciad428 37463564

[53] Tamma PD, Heil EL, Justo JA, Mathers AJ, Satlin MJ, Bonomo RA. 2024. Infectious Diseases Society of America 2024 guidance on the treatment of antimicrobial-resistant Gram-negative infections. Clin Infect Dis:ciae403. doi:10.1093/cid/ciae403 39108079

[54] Penwell WF, Shapiro AB, Giacobbe RA, Gu R-F, Gao N, Thresher J, McLaughlin RE, Huband MD, DeJonge BLM, Ehmann DE, Miller AA. 2015. Molecular mechanisms of sulbactam antibacterial activity and resistance determinants in Acinetobacter baumannii. Antimicrob Agents Chemother 59:1680–1689. doi:10.1128/AAC.04808-14 25561334 PMC4325763

[55] CLSI. 2024. Performance standards for antimicrobial susceptibility testing. In CLSI supplement M100, 34th ed. Clinical and Laboratory Standards Institute, USA.

[56] Yokoyama Y, Matsumoto K, Ikawa K, Watanabe E, Shigemi A, Umezaki Y, Nakamura K, Ueno K, Morikawa N, Takeda Y. 2014. Pharmacokinetic/pharmacodynamic evaluation of sulbactam against Acinetobacter baumannii in in vitro and murine thigh and lung infection models. Int J Antimicrob Agents 43:547–552. doi:10.1016/j.ijantimicag.2014.02.012 24796218

[57] Abouelhassan Y, Kuti JL, Nicolau DP, Abdelraouf K. 2024. Ampicillin-sulbactam against Acinetobacter baumannii infections: pharmacokinetic/pharmacodynamic appraisal of current susceptibility breakpoints and dosing recommendations. J Antimicrob Chemother 79:2227–2236. doi:10.1093/jac/dkae218 39031073

[58] Berry AV, Kuti JL. 2022. Pharmacodynamic thresholds for beta-lactam antibiotics: a story of mouse versus man. Front Pharmacol 13:833189. doi:10.3389/fphar.2022.833189 35370708 PMC8971958

[59] Makris D, Petinaki E, Tsolaki V, Manoulakas E, Mantzarlis K, Apostolopoulou O, Sfyras D, Zakynthinos E. 2018. Colistin versus colistin combined with ampicillin-sulbactam for multiresistant Acinetobacter baumannii ventilator-associated pneumonia treatment: an open-label prospective study. Indian J Crit Care Med 22:67–77. doi:10.4103/ijccm.IJCCM_302_17 29531445 PMC5842460

[60] Betrosian AP, Frantzeskaki F, Xanthaki A, Georgiadis G. 2007. High-dose ampicillin-sulbactam as an alternative treatment of late-onset VAP from multidrug-resistant Acinetobacter baumannii. Scand J Infect Dis 39:38–43. doi:10.1080/00365540600951184 17366011

[61] Jeong IB, Na MJ, Son JW, Jo DY, Kwon SJ. 2016. High-dose sulbactam treatment for ventilator-associated pneumonia caused by carbapenem-resistant Acinetobacter baumannii. Korean J Crit Care Med 31:308–316. doi:10.4266/kjccm.2015.00703

[62] Liu J, Shu Y, Zhu F, Feng B, Zhang Z, Liu L, Wang G. 2021. Comparative efficacy and safety of combination therapy with high-dose sulbactam or colistin with additional antibacterial agents for multiple drug-resistant and extensively drug-resistant Acinetobacter baumannii infections: a systematic review and network meta-analysis. J Glob Antimicrob Resist 24:136–147. doi:10.1016/j.jgar.2020.08.021 32889142

[63] Jung SY, Lee SH, Lee SY, Yang S, Noh H, Chung EK, Lee JI. 2017. Antimicrobials for the treatment of drug-resistant Acinetobacter baumannii pneumonia in critically ill patients: a systemic review and Bayesian network meta-analysis. Crit Care 21:319. doi:10.1186/s13054-017-1916-6 29262831 PMC5738897

[64] Khawcharoenporn T, Pruetpongpun N, Tiamsak P, Rutchanawech S, Mundy LM, Apisarnthanarak A. 2014. Colistin-based treatment for extensively drug-resistant Acinetobacter baumannii pneumonia. Int J Antimicrob Agents 43:378–382. doi:10.1016/j.ijantimicag.2014.01.016 24613422

[65] Ungthammakhun C, Vasikasin V, Changpradub D. 2019. Clinical outcomes of colistin in combination with either 6-G sulbactam or carbapenems for the treatment of extensively drug-resistant Acinetobacter baumannii pneumonia with high MIC to sulbactam, a prospective cohort study. Infect Drug Resist 12:2899–2904. doi:10.2147/IDR.S225518 31571943 PMC6750850

[66] Karageorgopoulos DE, Kelesidis T, Kelesidis I, Falagas ME. 2008. Tigecycline for the treatment of multidrug-resistant (including carbapenem-resistant) Acinetobacter infections: a review of the scientific evidence. Journal of Antimicrobial Chemotherapy 62:45–55. doi:10.1093/jac/dkn165 18436554 PMC8057304

[67] Bauer G, Berens C, Projan SJ, Hillen W. 2004. Comparison of tetracycline and tigecycline binding to ribosomes mapped by dimethylsulphate and drug-directed Fe2+ cleavage of 16S rRNA. J Antimicrob Chemother 53:592–599. doi:10.1093/jac/dkh125 14985271

[68] McGovern PC, Wible M, El-Tahtawy A, Biswas P, Meyer RD. 2013. All-cause mortality imbalance in the tigecycline phase 3 and 4 clinical trials. Int J Antimicrob Agents 41:463–467. doi:10.1016/j.ijantimicag.2013.01.020 23537581

[69] Wang JL, Lai CC, Ko WC, Hsueh PR. 2023. Geographical patterns of in vitro susceptibilities to tigecycline and colistin among worldwide isolates of Acinetobacter baumannii, Escherichia coli and Klebsiella pneumoniae: data from the Antimicrobial Testing Leadership and Surveillance (ATLAS) programme, 2016-2021. Int J Antimicrob Agents 62:106930. doi:10.1016/j.ijantimicag.2023.106930 37490959

[70] Xie J, Roberts JA, Alobaid AS, Roger C, Wang Y, Yang Q, Sun J, Dong H, Wang X, Xing J, Lipman J, Dong Y. 2017. Population pharmacokinetics of tigecycline in critically Ill patients with severe infections. Antimicrob Agents Chemother 61:e00345-17. doi:10.1128/AAC.00345-17 28607024 PMC5527570

[71] Luo X, Wang S, Li D, Wen J, Sun N, Fan G. 2023. Population pharmacokinetics of tigecycline in critically ill patients. Front Pharmacol 14:1083464. doi:10.3389/fphar.2023.1083464 36992827 PMC10040605

[72] De Pascale G, Lisi L, Ciotti GMP, Vallecoccia MS, Cutuli SL, Cascarano L, Gelormini C, Bello G, Montini L, Carelli S, Di Gravio V, Tumbarello M, Sanguinetti M, Navarra P, Antonelli M. 2020. Pharmacokinetics of high-dose tigecycline in critically ill patients with severe infections. Ann Intensive Care 10:94. doi:10.1186/s13613-020-00715-2 32661791 PMC7357259

[73] Liang CA, Lin YC, Lu PL, Chen HC, Chang HL, Sheu CC. 2018. Antibiotic strategies and clinical outcomes in critically ill patients with pneumonia caused by carbapenem-resistant Acinetobacter baumannii. Clin Microbiol Infect 24:908. doi:10.1016/j.cmi.2017.10.033 29108947

[74] Liou BH, Lee YT, Kuo SC, Liu PY, Fung CP. 2015. Efficacy of tigecycline for secondary Acinetobacter bacteremia and factors associated with treatment failure. Antimicrob Agents Chemother 59:3637–3640. doi:10.1128/AAC.04987-14 25824230 PMC4432115

[75] Niu T, Luo Q, Li Y, Zhou Y, Yu W, Xiao Y. 2019. Comparison of tigecycline or cefoperazone/sulbactam therapy for bloodstream infection due to carbapenem-resistant Acinetobacter baumannii. Antimicrob Resist Infect Control 8:52. doi:10.1186/s13756-019-0502-x 30886705 PMC6404342

[76] Zha L, Pan L, Guo J, French N, Villanueva EV, Tefsen B. 2020. Effectiveness and safety of high dose tigecycline for the treatment of severe infections: a systematic review and meta-analysis. Adv Ther 37:1049–1064. doi:10.1007/s12325-020-01235-y 32006240 PMC7223407

[77] Sun C, Yu Y, Hua X. 2023. Resistance mechanisms of tigecycline in Acinetobacter baumannii. Front Cell Infect Microbiol 13:1141490. doi:10.3389/fcimb.2023.1141490 37228666 PMC10203620

[78] Scheetz MH, Qi C, Warren JR, Postelnick MJ, Zembower T, Obias A, Noskin GA. 2007. In vitro activities of various antimicrobials alone and in combination with tigecycline against carbapenem-intermediate or -resistant Acinetobacter baumannii. Antimicrob Agents Chemother 51:1621–1626. doi:10.1128/AAC.01099-06 17307973 PMC1855572

[79] Pfaller MA, Shortridge D, Carvalhaes CG, Castanheira M. 2023. Trends in the susceptibility of U.S. Acinetobacter baumannii-calcoaceticus species complex and Stenotrophomonas maltophilia isolates to minocycline, 2014-2021. Microbiol Spectr 11:e0198123. doi:10.1128/spectrum.01981-23 37921464 PMC10715018

[80] Flamm RK, Shortridge D, Castanheira M, Sader HS, Pfaller MA. 2019. In vitro activity of minocycline against U.S. isolates of Acinetobacter baumannii-Acinetobacter calcoaceticus species complex, Stenotrophomonas maltophilia, and Burkholderia cepacia complex: results from the SENTRY Antimicrobial Surveillance Program, 2014 to 2018. Antimicrob Agents Chemother 63:e01154-19. doi:10.1128/AAC.01154-19 31427295 PMC6811394

[81] Greig SL, Scott LJ. 2016. Intravenous minocycline: a review in acinetobacter infections. Drugs (Abingdon Engl) 76:1467–1476. doi:10.1007/s40265-016-0636-6 27573640

[82] Lodise TP, Van Wart S, Sund ZM, Bressler AM, Khan A, Makley AT, Hamad Y, Salata RA, Silveira FP, Sims MD, Kabchi BA, Saad MA, Brown C, Oler RE Jr, Fowler V Jr, Wunderink RG. 2021. Pharmacokinetic and pharmacodynamic profiling of minocycline for injection following a single infusion in critically Ill adults in a phase IV open-label multicenter study (ACUMIN). Antimicrob Agents Chemother 65:e01809-20. doi:10.1128/AAC.01809-20 33168615 PMC8092545

[83] Fragkou PC, Poulakou G, Blizou A, Blizou M, Rapti V, Karageorgopoulos DE, Koulenti D, Papadopoulos A, Matthaiou DK, Tsiodras S. 2019. The role of minocycline in the treatment of nosocomial infections caused by multidrug, extensively drug and pandrug resistant Acinetobacter baumannii: a systematic review of clinical evidence. Microorganisms 7:159. doi:10.3390/microorganisms7060159 31159398 PMC6617316

[84] Lashinsky JN, Henig O, Pogue JM, Kaye KS. 2017. Minocycline for the treatment of multidrug and extensively drug-resistant A. baumannii: a review. Infect Dis Ther 6:199–211. doi:10.1007/s40121-017-0153-2 28357705 PMC5446366

[85] Goff DA, Bauer KA, Mangino JE. 2014. Bad bugs need old drugs: a stewardship program’s evaluation of minocycline for multidrug-resistant Acinetobacter baumannii infections. Clin Infect Dis 59 Suppl 6:S381–7. doi:10.1093/cid/ciu593 25371514

[86] Beganovic M, Daffinee KE, Luther MK, LaPlante KL. 2021. Minocycline alone and in combination with polymyxin B, meropenem, and sulbactam against carbapenem-susceptible and -resistant Acinetobacter baumannii in an in vitro pharmacodynamic model. Antimicrob Agents Chemother 65:e01680-20. doi:10.1128/AAC.01680-20 33318006 PMC8092495

[87] Rolston K, Gerges B, Nesher L, Shelburne SA, Prince R, Raad I. 2023. In vitro activity of eravacycline and comparator agents against bacterial pathogens isolated from patients with cancer. JAC Antimicrob Resist 5:dlad020. doi:10.1093/jacamr/dlad020 36875177 PMC9981869

[88] Zhanel GG, Cheung D, Adam H, Zelenitsky S, Golden A, Schweizer F, Gorityala B, Lagacé-Wiens PRS, Walkty A, Gin AS, Hoban DJ, Karlowsky JA. 2016. Review of eravacycline, a novel fluorocycline antibacterial agent. Drugs (Abingdon Engl) 76:567–588. doi:10.1007/s40265-016-0545-8 26863149

[89] Abdallah M, Olafisoye O, Cortes C, Urban C, Landman D, Quale J. 2015. Activity of eravacycline against Enterobacteriaceae and Acinetobacter baumannii, including multidrug-resistant isolates, from New York City. Antimicrob Agents Chemother 59:1802–1805. doi:10.1128/AAC.04809-14 25534744 PMC4325809

[90] Livermore DM, Mushtaq S, Warner M, Woodford N. 2016. In vitro activity of eravacycline against carbapenem-resistant Enterobacteriaceae and Acinetobacter baumannii. Antimicrob Agents Chemother 60:3840–3844. doi:10.1128/AAC.00436-16 27044556 PMC4879382

[91] Seifert H, Stefanik D, Sutcliffe JA, Higgins PG. 2018. In-vitro activity of the novel fluorocycline eravacycline against carbapenem non-susceptible Acinetobacter baumannii. Int J Antimicrob Agents 51:62–64. doi:10.1016/j.ijantimicag.2017.06.022 28705668

[92] Jing R, Yi QL, Zhuo C, Kang W, Yang QW, Yu YS, Zheng B, Li Y, Hu FP, Yang Y, Lin J, Zhang G, Zhang JJ, Wang T, Li J, Zhuo CY, Li X, Zhu YF, Xu YC. 2024. Establishment of epidemiological cut-off values for eravacycline, against Escherichia coli, Klebsiella pneumoniae, Enterobacter cloacae, Acinetobacter baumannii and Staphylococcus aureus. J Antimicrob Chemother 79:2246–2250. doi:10.1093/jac/dkae220 39011845 PMC11368425

[93] Alosaimy S, Morrisette T, Lagnf AM, Rojas LM, King MA, Pullinger BM, Hobbs ALV, Perkins NB III, Veve MP, Bouchard J, Gore T, Jones B, Truong J, Andrade J, Huang G, Cosimi R, Kang-Birken SL, Molina KC, Biagi M, Pierce M, Scipione MR, Zhao JJ, Davis SL, Rybak MJ. 2022. Clinical outcomes of eravacycline in patients treated predominately for carbapenem-resistant Acinetobacter baumannii. Microbiol Spectr 10:e0047922. doi:10.1128/spectrum.00479-22 36190427 PMC9602915

[94] Jackson MNW, Wei W, Mang NS, Prokesch BC, Ortwine JK. 2024. Combination eravacycline therapy for ventilator-associated pneumonia due to carbapenem-resistant Acinetobacter baumannii in patients with COVID-19: a case series. Pharmacotherapy 44:301–307. doi:10.1002/phar.2908 38270447

[95] Scott CJ, Zhu E, Jayakumar RA, Shan G, Viswesh V. 2022. Efficacy of eravacycline versus best previously available therapy for adults with pneumonia due to difficult-to-treat resistant (DTR) Acinetobacter baumannii. Ann Pharmacother 56:1299–1307. doi:10.1177/10600280221085551 35511209

[96] Solomkin JS, Gardovskis J, Lawrence K, Montravers P, Sway A, Evans D, Tsai L. 2019. IGNITE4: results of a phase 3, randomized, multicenter, prospective trial of eravacycline vs meropenem in the treatment of complicated intraabdominal infections. Clin Infect Dis 69:921–929. doi:10.1093/cid/ciy1029 30561562 PMC6735687

[97] Solomkin J, Evans D, Slepavicius A, Lee P, Marsh A, Tsai L, Sutcliffe JA, Horn P. 2017. Assessing the efficacy and safety of eravacycline vs ertapenem in complicated intra-abdominal infections in the investigating gram-negative infections treated with eravacycline (IGNITE 1) trial: a randomized clinical trial. JAMA Surg 152:224–232. doi:10.1001/jamasurg.2016.4237 27851857

[98] Clark RB, Hunt DK, He M, Achorn C, Chen C-L, Deng Y, Fyfe C, Grossman TH, Hogan PC, O’Brien WJ, Plamondon L, Rönn M, Sutcliffe JA, Zhu Z, Xiao X-Y. 2012. Fluorocyclines. 2. Optimization of the C-9 side-chain for antibacterial activity and oral efficacy. J Med Chem 55:606–622. doi:10.1021/jm201467r 22148555

[99] Beheshti M, Talebi M, Ardebili A, Bahador A, Lari AR. 2014. Detection of AdeABC efflux pump genes in tetracycline-resistant Acinetobacter baumannii isolates from burn and ventilator-associated pneumonia patients. J Pharm Bioall Sci 6:229. doi:10.4103/0975-7406.142949 PMC4231381 25400404

[100] Yamano Y. 2019. In vitro activity of cefiderocol against a broad range of clinically important Gram-negative bacteria. Clin Infect Dis 69:S544–S551. doi:10.1093/cid/ciz827 31724049 PMC6853761

[101] Karlowsky JA, Hackel MA, Takemura M, Yamano Y, Echols R, Sahm DF. 2022. In vitro susceptibility of Gram-negative pathogens to cefiderocol in five consecutive annual multinational SIDERO-WT surveillance studies, 2014 to 2019. Antimicrob Agents Chemother 66:e0199021. doi:10.1128/AAC.01990-21 34807757 PMC8846469

[102] Longshaw C, Manissero D, Tsuji M, Echols R, Yamano Y. 2020. In vitro activity of the siderophore cephalosporin, cefiderocol, against molecularly characterized, carbapenem-non-susceptible Gram-negative bacteria from Europe. JAC Antimicrob Resist 2:dlaa060. doi:10.1093/jacamr/dlaa060 34223017 PMC8210120

[103] Gill K, Takamichi B, Cooper A. 2024. Clinical efficacy of cefiderocol-based regimens in patients with carbapenem-resistant Acinetobacter baumannii infections: new data from CREDIBLE-CR with an updated meta-analysis. Int J Antimicrob Agents 63:107167. doi:10.1016/j.ijantimicag.2024.107167 38582402

[104] Gatti M, Cosentino F, Giannella M, Viale P, Pea F. 2024. Clinical efficacy of cefiderocol-based regimens in patients with carbapenem-resistant Acinetobacter baumannii infections: a systematic review with meta-analysis. Int J Antimicrob Agents 63:107047. doi:10.1016/j.ijantimicag.2023.107047 38061418

[105] Onorato L, de Luca I, Monari C, Coppola N. 2024. Cefiderocol either in monotherapy or combination versus best available therapy in the treatment of carbapenem-resistant Acinetobacter baumannii infections: a systematic review and meta-analysis. J Infect 88:106113. doi:10.1016/j.jinf.2024.01.012 38331328

[106] Zhan Y, Mao W, Zhao C, Lu D, Chen C, Hu W, Yang Q. 2024. Comparison of cefiderocol and colistin-based regimens for the treatment of severe infections caused by carbapenem-resistant Acinetobacter baumannii: a systematic review with meta-analysis and trial sequential analysis. BMC Infect Dis 24:967. doi:10.1186/s12879-024-09899-5 39271977 PMC11395218

[107] Clancy CJ, Cornely OA, Marcella SW, Nguyen ST, Gozalo L, Cai B. 2024. Effectiveness and safety of cefiderocol in clinical practice for treatment of patients with gram-negative bacterial infections: US Interim Results of the PROVE Study. Infect Drug Resist 17:4427–4443. doi:10.2147/IDR.S475462 39431212 PMC11490232

[108] Clancy N. 2024. Real-world effectiveness and safety of cefiderocol in the treatment of patients with serious Gram-negative bacterial infections: results of the PROVE chart review study. Poster presented at IDWeek 2024

[109] Shields RK, Dorazio AJ, Tiseo G, Squires KM, Leonildi A, Giordano C, Kline EG, Barnini S, Iovleva A, Griffith MP, Van Tyne D, Doi Y, Falcone M. 2024. Frequency of cefiderocol heteroresistance among patients treated with cefiderocol for carbapenem-resistant Acinetobacter baumannii infections. JAC Antimicrob Resist 6:dlae146. doi:10.1093/jacamr/dlae146 39253335 PMC11382143

[110] Longshaw C, Santerre Henriksen A, Dressel D, Malysa M, Silvestri C, Takemura M, Yamano Y, Baba T, Slover CM. 2023. Heteroresistance to cefiderocol in carbapenem-resistant Acinetobacter baumannii in the CREDIBLE-CR study was not linked to clinical outcomes: a post hoc analysis. Microbiol Spectr 11:e0237123. doi:10.1128/spectrum.02371-23 37966262 PMC10714777

[111] Choby JE, Ozturk T, Satola SW, Jacob JT, Weiss DS. 2021. Widespread cefiderocol heteroresistance in carbapenem-resistant Gram-negative pathogens. Lancet Infect Dis 21:597–598. doi:10.1016/S1473-3099(21)00194-8 PMC8175093 33894839

[112] Malik S, Kaminski M, Landman D, Quale J. 2020. Cefiderocol resistance in Acinetobacter baumannii: roles of beta-lactamases, siderophore receptors, and penicillin binding protein 3. Antimicrob Agents Chemother 64:e01221-20. doi:10.1128/AAC.01221-20 32868330 PMC7577126

[113] Asrat H, Samaroo-Campbell J, Ata S, Quale J. 2023. Contribution of iron-transport systems and β-lactamases to cefiderocol resistance in clinical isolates of Acinetobacter baumannii endemic to New York City. Antimicrob Agents Chemother 67:e0023423. doi:10.1128/aac.00234-23 37212653 PMC10269113

[114] Smoke SM, Brophy A, Reveron S, Iovleva A, Kline EG, Marano M, Miller LP, Shields RK. 2023. Evolution and transmission of cefiderocol-resistant Acinetobacter baumannii during an outbreak in the burn intensive care unit. Clin Infect Dis 76:e1261–e1265. doi:10.1093/cid/ciac647 35974429 PMC10169418

[115] Tiseo G, Giordano C, Leonildi A, Riccardi N, Galfo V, Limongi F, Nicastro M, Barnini S, Falcone M. 2023. Salvage therapy with sulbactam/durlobactam against cefiderocol-resistant Acinetobacter baumannii in a critically ill burn patient: clinical challenges and molecular characterization. JAC Antimicrob Resist 5:dlad078. doi:10.1093/jacamr/dlad078 37325251 PMC10265591

[116] Castanheira M, Deshpande LM, Mendes RE, Doyle TB, Sader HS. 2022. Prevalence of carbapenemase genes among carbapenem-nonsusceptible Enterobacterales collected in US hospitals in a five-year period and activity of ceftazidime/avibactam and comparator agents. JAC Antimicrob Resist 4:dlac098. doi:10.1093/jacamr/dlac098 36196444 PMC9524567

[117] Sader HS, Mendes RE, Carvalhaes CG, Kimbrough JH, Castanheira M. 2023. Changing epidemiology of carbapenemases among carbapenem-resistant enterobacterales from United States hospitals and the activity of aztreonam-avibactam against contemporary Enterobacterales (2019-2021). Open Forum Infect Dis 10:ofad046. doi:10.1093/ofid/ofad046 36846612 PMC9945928

[118] Sartor AL, Raza MW, Abbasi SA, Day KM, Perry JD, Paterson DL, Sidjabat HE. 2014. Molecular epidemiology of NDM-1-producing Enterobacteriaceae and Acinetobacter baumannii isolates from Pakistan. Antimicrob Agents Chemother 58:5589–5593. doi:10.1128/AAC.02425-14 24982081 PMC4135889

[119] Perry JD, Naqvi SH, Mirza IA, Alizai SA, Hussain A, Ghirardi S, Orenga S, Wilkinson K, Woodford N, Zhang J, Livermore DM, Abbasi SA, Raza MW. 2011. Prevalence of faecal carriage of Enterobacteriaceae with NDM-1 carbapenemase at military hospitals in Pakistan, and evaluation of two chromogenic media. J Antimicrob Chemother 66:2288–2294. doi:10.1093/jac/dkr299 21788293

[120] Heydari F, Mammina C, Koksal F. 2015. NDM-1-producing Acinetobacter baumannii ST85 now in Turkey, including one isolate from a Syrian refugee. J Med Microbiol 64:1027–1029. doi:10.1099/jmm.0.000132 26296677

[121] Kaase M, Nordmann P, Wichelhaus TA, Gatermann SG, Bonnin RA, Poirel L. 2011. NDM-2 carbapenemase in Acinetobacter baumannii from Egypt. J Antimicrob Chemother 66:1260–1262. doi:10.1093/jac/dkr135 21427107

[122] Bonnin RA, Poirel L, Naas T, Pirs M, Seme K, Schrenzel J, Nordmann P. 2012. Dissemination of New Delhi metallo-β-lactamase-1-producing Acinetobacter baumannii in Europe. Clin Microbiol Infect 18:E362–E365. doi:10.1111/j.1469-0691.2012.03928.x 22738206

[123] Gottesman T, Fedorowsky R, Yerushalmi R, Lellouche J, Nutman A. 2021. An outbreak of carbapenem-resistant Acinetobacter baumannii in a COVID-19 dedicated hospital. Infect Prev Pract 3:100113. doi:10.1016/j.infpip.2021.100113 34316574 PMC7794049

[124] Holden D, Mitsunaga T, Sanford D, Fryer T, Nash J, Schneider E, Mukhopadhyay R, Epson E, Sylvester M. 2021. Multifacility outbreak of NDM/OXA-23–producing Acinetobacter baumannii in California, 2020–2021. ASHE 1:s79–s79. doi:10.1017/ash.2021.155

[125] Adler A, Ghosh H, Gross A, Rechavi A, Lasnoy M, Assous MV, Geffen Y, Darawsha B, Wiener-Well Y, Alony A, Grundmann H, Reuter S. 2023. Clinical and molecular features of NDM-producing Acinetobacter baumannii in a multicenter study in Israel. Ann Clin Microbiol Antimicrob 22:52. doi:10.1186/s12941-023-00607-w 37391819 PMC10314562

[126] Poirel L, Sadek M, Nordmann P. 2021. Contribution of PER-type and NDM-type β-lactamases to cefiderocol resistance in Acinetobacter baumannii. Antimicrob Agents Chemother 65:e0087721. doi:10.1128/AAC.00877-21 34252309 PMC8448131

[127] El-Ghali A, Kunz Coyne AJ, Caniff K, Bleick C, Rybak MJ. 2023. Sulbactam-durlobactam: a novel β-lactam-β-lactamase inhibitor combination targeting carbapenem-resistant Acinetobacter baumannii infections. Pharmacotherapy 43:502–513. doi:10.1002/phar.2802 37052117

[128] Durand-Réville TF, Guler S, Comita-Prevoir J, Chen B, Bifulco N, Huynh H, Lahiri S, Shapiro AB, McLeod SM, Carter NM, et al.. 2017. ETX2514 is a broad-spectrum β-lactamase inhibitor for the treatment of drug-resistant Gram-negative bacteria including Acinetobacter baumannii. Nat Microbiol 2:17104. doi:10.1038/nmicrobiol.2017.104 28665414

[129] Barnes MD, Kumar V, Bethel CR, Moussa SH, O’Donnell J, Rutter JD, Good CE, Hujer KM, Hujer AM, Marshall SH, Kreiswirth BN, Richter SS, Rather PN, Jacobs MR, Papp-Wallace KM, van den Akker F, Bonomo RA. 2019. Targeting multidrug-resistant Acinetobacter spp.: sulbactam and the diazabicyclooctenone β-lactamase inhibitor ETX2514 as a novel therapeutic agent. MBio 10:e00159-19. doi:10.1128/mBio.00159-19 30862744 PMC6414696

[130] McLeod SM, Shapiro AB, Moussa SH, Johnstone M, McLaughlin RE, de Jonge BLM, Miller AA. 2018. Frequency and mechanism of spontaneous resistance to sulbactam combined with the novel β-lactamase inhibitor ETX2514 in clinical isolates of Acinetobacter baumannii. Antimicrob Agents Chemother 62:e01576-17. doi:10.1128/AAC.01576-17 29133555 PMC5786785

[131] Kaye KS, Shorr AF, Wunderink RG, Du B, Poirier GE, Rana K, Miller A, Lewis D, O’Donnell J, Chen L, Reinhart H, Srinivasan S, Isaacs R, Altarac D. 2023. Efficacy and safety of sulbactam–durlobactam versus colistin for the treatment of patients with serious infections caused by Acinetobacter baumannii–calcoaceticus complex: a multicentre, randomised, active-controlled, phase 3, non-inferiority clinical trial (ATTACK). Lancet Infect Dis 23:1072–1084. doi:10.1016/S1473-3099(23)00184-6 37182534

[132] Choi JY, Park YS, Cho CH, Park YS, Shin SY, Song YG, Yong D, Lee K, Kim JM. 2004. Synergic in-vitro activity of imipenem and sulbactam against Acinetobacter baumannii. Clin Microbiol Infect 10:1098–1101. doi:10.1111/j.1469-0691.2004.00987.x 15606639

[133] Fernández-Cuenca F, Martínez-Martínez L, Conejo MC, Ayala JA, Perea EJ, Pascual A. 2003. Relationship between beta-lactamase production, outer membrane protein and penicillin-binding protein profiles on the activity of carbapenems against clinical isolates of Acinetobacter baumannii. J Antimicrob Chemother 51:565–574. doi:10.1093/jac/dkg097 12615856

[134] Miller A, Tommasi R. 2022. Sulbactam-durlobactam (SUL-DUR) in vitro dose response studies with and without imipenem or meropenem carbapenemase-producing Acinetobacter baumannii utilizing the hollow-fiber infection model, abstr ECCMID, Lisbon, Portugal

[135] Holger DJ, Kunz Coyne AJ, Zhao JJ, Sandhu A, Salimnia H, Rybak MJ. 2022. Novel combination therapy for extensively drug-resistant Acinetobacter baumannii necrotizing pneumonia complicated by empyema: a case report. Open Forum Infect Dis 9:ofac092. doi:10.1093/ofid/ofac092 35350174 PMC8946682

[136] O’Donnell J, Tanudra A, Chen A, Miller AA, McLeod SM, Tommasi R. 2024. In vitro pharmacokinetics/pharmacodynamics of the beta-lactamase inhibitor, durlobactam, in combination with sulbactam against Acinetobacter baumannii-calcoaceticus complex. Antimicrob Agents Chemother 68:e0031223. doi:10.1128/aac.00312-23 38092676 PMC10869334

[137] McLeod S, Carter N, Moussa S, Miller A. 2023. 2138. In vitro susceptibility of recent clinical isolates of P. aeruginosa and Enterobacterales to imipenem or meropenem alone or in combination with sulbactam-durlobactam. Open Forum Infect Dis 10. doi:10.1093/ofid/ofad500.1761

[138] Tamma PD, Immel S, Karaba SM, Soto CL, Conzemius R, Gisriel E, Tekle T, Stambaugh H, Johnson E, Tornheim JA, Simner PJ. 2024. Successful treatment of carbapenem-resistant Acinetobacter baumannii meningitis with sulbactam-durlobactam. Clin Infect Dis 79:819–825. doi:10.1093/cid/ciae210 38630890 PMC11478584

[139] McLeod SM, Carter NM, Bradford PA, Miller AA. 2024. In vitro antibacterial activity of sulbactam-durlobactam in combination with other antimicrobial agents against Acinetobacter baumannii-calcoaceticus complex. Diagn Microbiol Infect Dis 109:116344. doi:10.1016/j.diagmicrobio.2024.116344 38735147

[140] Zaidan N, Hornak JP, Reynoso D. 2021. Extensively drug-resistant Acinetobacter baumannii nosocomial pneumonia successfully treated with a novel antibiotic combination. Antimicrob Agents Chemother 65:e0092421. doi:10.1128/AAC.00924-21 34370576 PMC8522738

[141] McLeod SM, Miller AA, Rana K, Altarac D, Moussa SH, Shapiro AB. 2024. Clinical outcomes for patients with monomicrobial vs polymicrobial Acinetobacter baumannii-calcoaceticus complex infections treated with sulbactam-durlobactam or colistin: a subset analysis from a phase 3 clinical trial. Open Forum Infect Dis 11:ofae140. doi:10.1093/ofid/ofae140 38595956 PMC11002948

[142] Fishbain J, Peleg AY. 2010. Treatment of Acinetobacter infections. Clin Infect Dis 51:79–84. doi:10.1086/653120 20504234

[143] CLSI. 2020. Polymyxin breakpoints for Enterobacterales, Pseudomonas aeruginosa, and Acinetobacter spp. 2nd ed. Clinical and Laboratory Standards Institute, Wayne, PA.

[144] Jeannot K, Bolard A, Plésiat P. 2017. Resistance to polymyxins in Gram-negative organisms. Int J Antimicrob Agents 49:526–535. doi:10.1016/j.ijantimicag.2016.11.029 28163137

[145] Paul M, Daikos GL, Durante-Mangoni E, Yahav D, Carmeli Y, Benattar YD, Skiada A, Andini R, Eliakim-Raz N, Nutman A, et al.. 2018. Colistin alone versus colistin plus meropenem for treatment of severe infections caused by carbapenem-resistant Gram-negative bacteria: an open-label, randomised controlled trial. Lancet Infect Dis 18:391–400. doi:10.1016/S1473-3099(18)30099-9 29456043

[146] Kaye KS, Marchaim D, Thamlikitkul V, Carmeli Y, Chiu C-H, Daikos G, Dhar S, Durante-Mangoni E, Gikas A, Kotanidou A, et al.. 2023. Colistin monotherapy versus combination therapy for carbapenem-resistant organisms. NEJM Evid 2. doi:10.1056/evidoa2200131 PMC10398788 37538951

[147] Durante-Mangoni E, Signoriello G, Andini R, Mattei A, De Cristoforo M, Murino P, Bassetti M, Malacarne P, Petrosillo N, Galdieri N, Mocavero P, Corcione A, Viscoli C, Zarrilli R, Gallo C, Utili R. 2013. Colistin and rifampicin compared with colistin alone for the treatment of serious infections due to extensively drug-resistant Acinetobacter baumannii: a multicenter, randomized clinical trial. Clin Infect Dis 57:349–358. doi:10.1093/cid/cit253 23616495

[148] Aydemir H, Akduman D, Piskin N, Comert F, Horuz E, Terzi A, Kokturk F, Ornek T, Celebi G. 2013. Colistin vs. the combination of colistin and rifampicin for the treatment of carbapenem-resistant Acinetobacter baumannii ventilator-associated pneumonia. Epidemiol Infect 141:1214–1222. doi:10.1017/S095026881200194X 22954403 PMC9151808

[149] Park HJ, Cho JH, Kim HJ, Han SH, Jeong SH, Byun MK. 2019. Colistin monotherapy versus colistin/rifampicin combination therapy in pneumonia caused by colistin-resistant Acinetobacter baumannii: a randomised controlled trial. J Glob Antimicrob Resist 17:66–71. doi:10.1016/j.jgar.2018.11.016 30476654

[150] Boisson M, Jacobs M, Grégoire N, Gobin P, Marchand S, Couet W, Mimoz O. 2014. Comparison of intrapulmonary and systemic pharmacokinetics of colistin methanesulfonate (CMS) and colistin after aerosol delivery and intravenous administration of CMS in critically ill patients. Antimicrob Agents Chemother 58:7331–7339. doi:10.1128/AAC.03510-14 25267660 PMC4249558

[151] Imberti R, Cusato M, Villani P, Carnevale L, Iotti GA, Langer M, Regazzi M. 2010. Steady-state pharmacokinetics and BAL concentration of colistin in critically Ill patients after IV colistin methanesulfonate administration. Chest 138:1333–1339. doi:10.1378/chest.10-0463 20558557

[152] Ardebili A, Izanloo A, Rastegar M. 2023. Polymyxin combination therapy for multidrug-resistant, extensively-drug resistant, and difficult-to-treat drug-resistant gram-negative infections: is it superior to polymyxin monotherapy? Expert Rev Anti Infect Ther 21:387–429. doi:10.1080/14787210.2023.2184346 36820511

[153] Rigatto MH, Vieira FJ, Antochevis LC, Behle TF, Lopes NT, Zavascki AP. 2015. Polymyxin B in combination with antimicrobials lacking in vitro activity versus Polymyxin B in monotherapy in critically Ill patients with Acinetobacter baumannii or Pseudomonas aeruginosa infections. Antimicrob Agents Chemother 59:6575–6580. doi:10.1128/AAC.00494-15 26259799 PMC4576098

[154] Rafailidis P, Panagopoulos P, Koutserimpas C, Samonis G. 2024. Current therapeutic approaches for multidrug-resistant and extensively drug-resistant Acinetobacter baumannii infections. Antibiotics (Basel) 13:261. doi:10.3390/antibiotics13030261 38534696 PMC10967523

[155] Adams MD, Nickel GC, Bajaksouzian S, Lavender H, Murthy AR, Jacobs MR, Bonomo RA. 2009. Resistance to colistin in Acinetobacter baumannii associated with mutations in the PmrAB two-component system. Antimicrob Agents Chemother 53:3628–3634. doi:10.1128/AAC.00284-09 19528270 PMC2737849

[156] Tao Y, Acket S, Beaumont E, Galez H, Duma L, Rossez Y. 2021. Colistin treatment affects lipid composition of Acinetobacter baumannii. Antibiotics (Basel) 10:528. doi:10.3390/antibiotics10050528 34063718 PMC8147793

[157] Ko KS, Suh JY, Kwon KT, Jung SI, Park KH, Kang CI, Chung DR, Peck KR, Song JH. 2007. High rates of resistance to colistin and polymyxin B in subgroups of Acinetobacter baumannii isolates from Korea. Journal of Antimicrobial Chemotherapy 60:1163–1167. doi:10.1093/jac/dkm305 17761499

[158] Jellison TK, Mckinnon PS, Rybak MJ. 2001. Epidemiology, resistance, and outcomes of Acinetobacter baumannii bacteremia treated with imipenem-cilastatin or ampicillin-sulbactam. Pharmacotherapy 21:142–148. doi:10.1592/phco.21.2.142.34114 11213849

[159] Wood GC, Hanes SD, Croce MA, Fabian TC, Boucher BA. 2002. Comparison of ampicillin-sulbactam and imipenem-cilastatin for the treatment of acinetobacter ventilator-associated pneumonia. Clin Infect Dis 34:1425–1430. doi:10.1086/340055 12015687

[160] Urban C, Segal-Maurer S, Rahal JJ. 2003. Considerations in control and treatment of nosocomial infections due to multidrug-resistant Acinetobacter baumannii. Clin Infect Dis 36:1268–1274. doi:10.1086/374847 12746772

[161] Abdul-Mutakabbir JC, Griffith NC, Shields RK, Tverdek FP, Escobar ZK. 2021. Contemporary perspective on the treatment of Acinetobacter baumannii infections: insights from the society of infectious diseases pharmacists. Infect Dis Ther 10:2177–2202. doi:10.1007/s40121-021-00541-4 34648177 PMC8514811

[162] Chang YY, Yang YS, Wu SL, Wang YC, Chen TL, Lee YT, Group AS. 2020. Comparison of cefepime-cefpirome and carbapenem therapy for Acinetobacter bloodstream infection in a multicenter study. Antimicrob Agents Chemother 64:e02392-19. doi:10.1128/AAC.02392-19 32179523 PMC7269511

[163] Higgins PG, Wisplinghoff H, Stefanik D, Seifert H. 2004. In vitro activities of the beta-lactamase inhibitors clavulanic acid, sulbactam, and tazobactam alone or in combination with beta-lactams against epidemiologically characterized multidrug-resistant Acinetobacter baumannii strains. Antimicrob Agents Chemother 48:1586–1592. doi:10.1128/AAC.48.5.1586-1592.2004 15105109 PMC400525

[164] Lesho E, Wortmann G, Moran K, Craft D. 2005. Fatal Acinetobacter baumannii infection with discordant carbapenem susceptibility. Clin Infect Dis 41:758–759. doi:10.1086/432623 16080102

[165] Wunderink RG, Matsunaga Y, Ariyasu M, Clevenbergh P, Echols R, Kaye KS, Kollef M, Menon A, Pogue JM, Shorr AF, Timsit JF, Zeitlinger M, Nagata TD. 2021. Cefiderocol versus high-dose, extended-infusion meropenem for the treatment of Gram-negative nosocomial pneumonia (APEKS-NP): a randomised, double-blind, phase 3, non-inferiority trial. Lancet Infect Dis 21:213–225. doi:10.1016/S1473-3099(20)30731-3 33058798

[166] Hänninen P, Rossi T. 1986. Penetration of sulbactam into cerebrospinal fluid of patients with viral meningitis or without meningitis. Rev Infect Dis 8 Suppl 5:S609–S611. doi:10.1093/clinids/8.supplement_5.s609 3026012

[167] Valcke YJ, Rosseel MT, Pauwels RA, Bogaert MG, Van der Straeten ME. 1990. Penetration of ampicillin and sulbactam in the lower airways during respiratory infections. Antimicrob Agents Chemother 34:958–962. doi:10.1128/AAC.34.6.958 2393293 PMC171737

[168] Jiménez-Mejías ME, Pachón J, Becerril B, Palomino-Nicás J, Rodríguez-Cobacho A, Revuelta M. 1997. Treatment of multidrug-resistant Acinetobacter baumannii meningitis with ampicillin/sulbactam. Clin Infect Dis 24:932–935. doi:10.1093/clinids/24.5.932 9142795

[169] Sun L, Wang X, Li Z. 2018. Successful treatment of multidrug-resistant Acinetobacter baumannii meningitis with ampicillin sulbactam in primary hospital. Br J Neurosurg 32:642–645. doi:10.1080/02688697.2017.1319907 28431478

[170] Levin AS, Levy CE, Manrique AEI, Medeiros EAS, Costa SF. 2003. Severe nosocomial infections with imipenem-resistant Acinetobacter baumannii treated with ampicillin/sulbactam. Int J Antimicrob Agents 21:58–62. doi:10.1016/S0924-8579(02)00276-5 12507838

[171] Pallotto C, Fiorio M, D’Avolio A, Sgrelli A, Baldelli F, Di Perri G, De Socio GV. 2014. Cerebrospinal fluid penetration of tigecycline. Scand J Infect Dis 46:69–72. doi:10.3109/00365548.2013.837957 24131423

[172] Huang Q, Zhang X, Jia A, Huang Q, Jiang Y, Xie L. 2022. Pharmacokinetics/pharmacodynamics and neurotoxicity of tigecycline intraventricular injection for the treatment of extensively drug-resistant Acinetobacter baumannii intracranial infection. Infect Drug Resist 15:4809–4817. doi:10.2147/IDR.S377772 36043158 PMC9420438

[173] Li Z, An Y, Li L, Yi H. 2022. Intrathecal injection of tigecycline and polymyxin B in the treatment of extensively drug-resistant intracranial Acinetobacter baumannii infection: a case report and review of the literature. Infect Drug Resist 15:1411–1423. doi:10.2147/IDR.S354460 35392365 PMC8980296

[174] Sanlıdağ Işbilen G, Akyol D, Yurtseven T, Ozgiray E, Cağlı MS, Aydemir S, Arda B, Sipahi OR. 2024. Intrathecal tigecycline in the treatment of hospital-acquired meningitis: a review of four cases. Surg Infect (Larchmt) 25:627–631. doi:10.1089/sur.2024.063 39056120

[175] Freire AT, Melnyk V, Kim MJ, Datsenko O, Dzyublik O, Glumcher F, Chuang YC, Maroko RT, Dukart G, Cooper CA, Korth-Bradley JM, Dartois N, Gandjini H, Study G. 2010. Comparison of tigecycline with imipenem/cilastatin for the treatment of hospital-acquired pneumonia. Diagn Microbiol Infect Dis 68:140–151. doi:10.1016/j.diagmicrobio.2010.05.012 20846586

[176] FDA. 2010. FDA drug safety communication: increased risk of death with tygacil (Tigecycline) compared to other antibiotics used to treat similar infections. Available from: https://www.fda.gov/drugs/drug-safety-and-availability/fda-drug-safety-communication-increased-risk-death-tygacil-tigecycline-compared-other-antibiotics

[177] Petersen PJ, Labthavikul P, Jones CH, Bradford PA. 2006. In vitro antibacterial activities of tigecycline in combination with other antimicrobial agents determined by chequerboard and time-kill kinetic analysis. J Antimicrob Chemother 57:573–576. doi:10.1093/jac/dki477 16431863

[178] Sopirala MM, Mangino JE, Gebreyes WA, Biller B, Bannerman T, Balada-Llasat JM, Pancholi P. 2010. Synergy testing by Etest, microdilution checkerboard, and time-kill methods for pan-drug-resistant Acinetobacter baumannii. Antimicrob Agents Chemother 54:4678–4683. doi:10.1128/AAC.00497-10 20713678 PMC2976112

[179] Ni W, Han Y, Zhao J, Wei C, Cui J, Wang R, Liu Y. 2016. Tigecycline treatment experience against multidrug-resistant Acinetobacter baumannii infections: a systematic review and meta-analysis. Int J Antimicrob Agents 47:107–116. doi:10.1016/j.ijantimicag.2015.11.011 26742726

[180] Saivin S, Houin G. 1988. Clinical pharmacokinetics of doxycycline and minocycline. Clin Pharmacokinet 15:355–366. doi:10.2165/00003088-198815060-00001 3072140

[181] Watanabe A, Anzai Y, Niitsuma K, Saito M, Yanase K, Nakamura M. 2001. Penetration of minocycline hydrochloride into lung tissue and sputum. Chemotherapy 47:1–9. doi:10.1159/000048494 11125226

[182] Zhou J, Ledesma KR, Chang KT, Abodakpi H, Gao S, Tam VH. 2017. Pharmacokinetics and pharmacodynamics of minocycline against Acinetobacter baumannii in a neutropenic murine pneumonia model. Antimicrob Agents Chemother 61:e02371-16. doi:10.1128/AAC.02371-16 28264853 PMC5404596

[183] Snowdin JW, Mercuro NJ, Madaio MP, Rawlings SA. 2024. Case report: successful treatment of OXA-23 Acinetobacter baumannii neurosurgical infection and meningitis with sulbactam-durlobactam combination therapy. Front Med 11:1381123. doi:10.3389/fmed.2024.1381123 PMC11135601 38813376

[184] Marcelo C, de Gea Grela A, Palazuelos MM, Veganzones J, Grandioso D, Díaz-Pollán B. 2022. Clinical cure of a difficult-to-treat resistant Pseudomonas aeruginosa ventriculitis using cefiderocol: a case report and literature review. Open Forum Infect Dis 9:ofac391. doi:10.1093/ofid/ofac391 35983267 PMC9379813

[185] Kufel WD, Abouelhassan Y, Steele JM, Gutierrez RL, Perwez T, Bourdages G, Nicolau DP. 2022. Plasma and cerebrospinal fluid concentrations of cefiderocol during successful treatment of carbapenem-resistant Acinetobacter baumannii meningitis. J Antimicrob Chemother 77:2737–2741. doi:10.1093/jac/dkac248 35869778

[186] Katsube T, Saisho Y, Shimada J, Furuie H. 2019. Intrapulmonary pharmacokinetics of cefiderocol, a novel siderophore cephalosporin, in healthy adult subjects. J Antimicrob Chemother 74:1971–1974. doi:10.1093/jac/dkz123 31220260 PMC6587409

[187] Finch NA, Granillo A, Pouya N, Bhimraj A, Miller WR, Tam VH. 2025. Pharmacokinetics of cefiderocol in a patient with carbapenem-resistant Acinetobacter baumannii ventriculitis: a case report. Pharmacotherapy 45:66–69. doi:10.1002/phar.4632 39629915 PMC11757030

[188] Kobic E, Abouelhassan Y, Singaravelu K, Nicolau DP. 2022. Pharmacokinetic analysis and in vitro synergy evaluation of cefiderocol, sulbactam, and tigecycline in an extensively drug-resistant Acinetobacter baumannii pneumonia patient receiving continuous venovenous hemodiafiltration. Open Forum Infect Dis 9:ofac484. doi:10.1093/ofid/ofac484 36225744 PMC9547507

[189] Cammarata AP, Safir MC, Trang M, Larson KB, O’Donnell JP, Bhavnani SM, Rubino CM. 2025. Population pharmacokinetic analyses for sulbactam-durlobactam using Phase 1, 2, and 3 data. Antimicrob Agents Chemother 69:e0048524. doi:10.1128/aac.00485-24 39569973 PMC11784299

[190] Petraitis V, Petraitiene R, Maung BBW, Khan F, Alisauskaite I, Olesky M, Newman J, Mutlib A, Niu X, Satlin M, Singh RS, Derendorf H, Walsh TJ. 2018. Pharmacokinetics and comprehensive analysis of the tissue distribution of eravacycline in rabbits. Antimicrob Agents Chemother 62:e00275-18. doi:10.1128/AAC.00275-18 29941646 PMC6125520

[191] Connors KP, Housman ST, Pope JS, Russomanno J, Salerno E, Shore E, Redican S, Nicolau DP. 2014. Phase I, open-label, safety and pharmacokinetic study to assess bronchopulmonary disposition of intravenous eravacycline in healthy men and women. Antimicrob Agents Chemother 58:2113–2118. doi:10.1128/AAC.02036-13 24468780 PMC4023791

[192] Lim A, Guo Y, McSweeney TD. 2023. 2137. Real world experience with eravacycline for the treatment of infections caused by carbapenem-resistant Acinetobacter baumannii. Open Forum Infect Dis 10. doi:10.1093/ofid/ofad500.1760

[193] Ozger HS, Cuhadar T, Yildiz SS, Demirbas Gulmez Z, Dizbay M, Guzel Tunccan O, Kalkanci A, Simsek H, Unaldi O. 2019. In vitro activity of eravacycline in combination with colistin against carbapenem-resistant A. baumannii isolates. J Antibiot 72:600–604. doi:10.1038/s41429-019-0188-6 31028352

[194] Foulds G, Stankewich JP, Marshall DC, O’Brien MM, Hayes SL, Weidler DJ, McMahon FG. 1983. Pharmacokinetics of sulbactam in humans. Antimicrob Agents Chemother 23:692–699. doi:10.1128/AAC.23.5.692 6307133 PMC184789

[195] Anonymous. 2023. Xacduro [package insert]. La Jolla Pharmaceutical Company, Waltham, MA.

[196] Anonymous. 2024. Fetroja [package insert]. Shionogi & Co., Ltd. , Osaka, Japan.

[197] Zhanel GG, Homenuik K, Nichol K, Noreddin A, Vercaigne L, Embil J, Gin A, Karlowsky JA, Hoban DJ. 2004. The glycylcyclines: a comparative review with the tetracyclines. Drugs (Abingdon Engl) 64:63–88. 10.2165/00003495-200464010-00005 14723559

[198] Anonymous. 2018. Eravacycline [package insert]. Watertown, MA.

[199] Liu YX, Le KJ, Shi HY, Zhang ZL, Cui M, Zhong H, Yu YT, Gu ZC. 2021. Efficacy and safety of tigecycline for complicated urinary tract infection: a systematic review. Transl Androl Urol 10:292–299. doi:10.21037/tau-20-959 33532318 PMC7844507

[200] Agwuh KN, MacGowan A. 2006. Pharmacokinetics and pharmacodynamics of the tetracyclines including glycylcyclines. J Antimicrob Chemother 58:256–265. doi:10.1093/jac/dkl224 16816396

[201] Anonymous. 2018. Tetraphase announces top-line results from IGNITE3 phase 3 clinical trial of eravacycline in complicated urinary tract infections (CUTI), on Tetraphase Pharmaceuticals, Inc. Available from: https://ir.tphase.com/news-releases/news-release-details/tetraphase-announces-top-line-results-ignite3-phase-3-clinical

[202] Tsuji BT, Pogue JM, Zavascki AP, Paul M, Daikos GL, Forrest A, Giacobbe DR, Viscoli C, Giamarellou H, Karaiskos I, Kaye D, Mouton JW, Tam VH, Thamlikitkul V, Wunderink RG, Li J, Nation RL, Kaye KS. 2019. International Consensus Guidelines for the Optimal Use of the Polymyxins: Endorsed by the American College of Clinical Pharmacy (ACCP), European Society of Clinical Microbiology and Infectious Diseases (ESCMID), Infectious Diseases Society of America (IDSA), International Society for Anti-infective Pharmacology (ISAP), Society of Critical Care Medicine (SCCM), and Society of Infectious Diseases Pharmacists (SIDP). Pharmacotherapy 39:10–39. doi:10.1002/phar.2209 30710469 PMC7437259

[203] Sandri AM, Landersdorfer CB, Jacob J, Boniatti MM, Dalarosa MG, Falci DR, Behle TF, Bordinhão RC, Wang J, Forrest A, Nation RL, Li J, Zavascki AP. 2013. Population pharmacokinetics of intravenous polymyxin B in critically ill patients: implications for selection of dosage regimens. Clin Infect Dis 57:524–531. doi:10.1093/cid/cit334 23697744

[204] Muralidharan G, Micalizzi M, Speth J, Raible D, Troy S. 2005. Pharmacokinetics of tigecycline after single and multiple doses in healthy subjects. Antimicrob Agents Chemother 49:220–229. doi:10.1128/AAC.49.1.220-229.2005 15616299 PMC538906

[205] Rodvold KA, Gotfried MH, Cwik M, Korth-Bradley JM, Dukart G, Ellis-Grosse EJ. 2006. Serum, tissue and body fluid concentrations of tigecycline after a single 100 mg dose. J Antimicrob Chemother 58:1221–1229. doi:10.1093/jac/dkl403 17012300

[206] Lee YT, Wang YC, Kuo SC, Chen CT, Liu CP, Liu YM, Chen TL, Yang YS. 2017. Multicenter study of clinical features of breakthrough Acinetobacter bacteremia during carbapenem therapy. Antimicrob Agents Chemother 61:e00931-17. doi:10.1128/AAC.00931-17 28674056 PMC5571354

[207] Kim NH, Hwang JH, Song KH, Choe PG, Kim ES, Park SW, Kim HB, Kim NJ, Park WB, Oh MD. 2013. Tigecycline in carbapenem-resistant Acinetobacter baumannii bacteraemia: susceptibility and clinical outcome. Scand J Infect Dis 45:315–319. doi:10.3109/00365548.2012.732705 23113680

[208] Anonymous. 2010. Minocin [package insert]. Triax Pharmaceuticals, LLC, Cincinnati, OH.

[209] Hobbs ALV, Gelfand MS, Cleveland KO, Saddler K, Sierra-Hoffman MA. 2022. A retrospective, multicentre evaluation of eravacycline utilisation in community and academic hospitals. J Glob Antimicrob Resist 29:430–433. doi:10.1016/j.jgar.2021.10.020 34788691

[210] Felice V-D, Efimova E, Izmailyan S, Napolitano LM, Chopra T. 2021. Efficacy and tolerability of eravacycline in bacteremic patients with complicated intra-abdominal infection: a pooled analysis from the IGNITE1 and IGNITE4 studies. Surg Infect (Larchmt) 22:556–561. doi:10.1089/sur.2020.241 33201771

[211] Gatti M, Bartoletti M, Cojutti PG, Gaibani P, Conti M, Giannella M, Viale P, Pea F. 2021. A descriptive case series of pharmacokinetic/pharmacodynamic target attainment and microbiological outcome in critically ill patients with documented severe extensively drug-resistant Acinetobacter baumannii bloodstream infection and/or ventilator-associated pneumonia treated with cefiderocol. J Glob Antimicrob Resist 27:294–298. doi:10.1016/j.jgar.2021.10.014 34710630

[212] Cheah S-E, Wang J, Nguyen VTT, Turnidge JD, Li J, Nation RL. 2015 New pharmacokinetic/pharmacodynamic studies of systemically administered colistin against Pseudomonas aeruginosa and Acinetobacter baumannii in mouse thigh and lung infection models: smaller response in lung infection. J Antimicrob Chemother:dkv267. doi:10.1093/jac/dkv267 26318190

[213] Landersdorfer CB, Wang J, Wirth V, Chen K, Kaye KS, Tsuji BT, Li J, Nation RL. 2018. Pharmacokinetics/pharmacodynamics of systemically administered polymyxin B against Klebsiella pneumoniae in mouse thigh and lung infection models. J Antimicrob Chemother 73:462–468. doi:10.1093/jac/dkx409 29149294 PMC5890666

[214] Zhang X, Cui X, Jiang M, Huang S, Yang M. 2023. Nebulized colistin as the adjunctive treatment for ventilator-associated pneumonia: a systematic review and meta-analysis. J Crit Care 77:154315. doi:10.1016/j.jcrc.2023.154315 37120926

[215] Karaiskos I, Gkoufa A, Polyzou E, Schinas G, Athanassa Z, Akinosoglou K. 2023. High-dose nebulized colistin methanesulfonate and the role in hospital-acquired pneumonia caused by Gram-negative bacteria with difficult-to-treat resistance: a review. Microorganisms 11:1459. doi:10.3390/microorganisms11061459 37374959 PMC10304325

[216] Ghosh S. 2024. Polymyxin B plus aerosolized colistin vs polymyxin B alone in hospital-acquired pneumonia (“AEROCOL” Study): a feasibility study. Indian J Crit Care Med 28:792–795. doi:10.5005/jp-journals-10071-24767 39239172 PMC11372667

[217] Tunkel AR, Hasbun R, Bhimraj A, Byers K, Kaplan SL, Scheld WM, van de Beek D, Bleck TP, Garton HJL, Zunt JR. 2017. 2017 Infectious Diseases Society of America’s clinical practice guidelines for healthcare-associated ventriculitis and meningitis. Clin Infect Dis 64:e34–e65. doi:10.1093/cid/ciw861 28203777 PMC5848239

[218] Kim BN, Peleg AY, Lodise TP, Lipman J, Li J, Nation R, Paterson DL. 2009. Management of meningitis due to antibiotic-resistant Acinetobacter species. Lancet Infect Dis 9:245–255. doi:10.1016/S1473-3099(09)70055-6 19324297 PMC2760093

[219] Lodise TP, Nau R, Kinzig M, Drusano GL, Jones RN, Sorgel F. 2007. Pharmacodynamics of ceftazidime and meropenem in cerebrospinal fluid: results of population pharmacokinetic modelling and Monte Carlo simulation. Journal of Antimicrobial Chemotherapy 60:1038–1044. doi:10.1093/jac/dkm325 17785282

[220] Stahl JP, Bru JP, Fredj G, Brammer KW, Malleret MR, Micoud M. 1986. Penetration of sulbactam into the cerebrospinal fluid of patients with bacterial meningitis receiving ampicillin therapy. Rev Infect Dis 8 Suppl 5:S612–6. doi:10.1093/clinids/8.supplement_5.s612 3026013

[221] Sollima A, Rossini F, Lanza P, Pallotto C, Meschiari M, Gentile I, Stellini R, Lenzi A, Mulé A, Castagna F, Lorenzotti S, Amadasi S, Van Hauwermeiren E, Saccani B, Fumarola B, Signorini L, Castelli F, Matteelli A. 2024. Role of cefiderocol in multidrug-resistant Gram-negative central nervous system infections: real life experience and state-of-the-art. Antibiotics (Basel) 13:453. doi:10.3390/antibiotics13050453 38786181 PMC11118811

[222] Kizilates F, Keskin AS, Onder KD. 2021. Clinical features of post-operative nosocomial meningitis in adults and evaluation of efficiency of intrathecal treatment. Surg Infect (Larchmt) 22:1059–1063. doi:10.1089/sur.2021.024 34352189

[223] Falagas ME, Bliziotis IA, Tam VH. 2007. Intraventricular or intrathecal use of polymyxins in patients with Gram-negative meningitis: a systematic review of the available evidence. Int J Antimicrob Agents 29:9–25. doi:10.1016/j.ijantimicag.2006.08.024 17126534

[224] Rodríguez Guardado A, Blanco A, Asensi V, Pérez F, Rial JC, Pintado V, Bustillo E, Lantero M, Tenza E, Alvarez M, Maradona JA, Cartón JA. 2008. Multidrug-resistant Acinetobacter meningitis in neurosurgical patients with intraventricular catheters: assessment of different treatments. J Antimicrob Chemother 61:908–913. doi:10.1093/jac/dkn018 18281693

[225] Cascio A, Conti A, Sinardi L, Iaria C, Angileri FF, Stassi G, David T, Versaci A, Iaria M, David A. 2010. Post-neurosurgical multidrug-resistant Acinetobacter baumannii meningitis successfully treated with intrathecal colistin. A new case and a systematic review of the literature. Int J Infect Dis 14:e572–e579. doi:10.1016/j.ijid.2009.06.032 19892577

[226] Rahal JJ, Hyams PJ, Simberkoff MS, Rubinstein E. 1974. Combined intrathecal and intramuscular gentamicin for gram-negative meningitis. Pharmacologic study of 21 patients. N Engl J Med 290:1394–1398. doi:10.1056/NEJM197406202902502 4208369

[227] Wright PF, Kaiser AB, Bowman CM, McKee KT, Trujillo H, McGee ZA. 1981. The pharmacokinetics and efficacy of an aminoglycoside administered into the cerebral ventricles in neonates: implications for further evaluation of this route of therapy in meningitis. J Infect Dis 143:141–147. doi:10.1093/infdis/143.2.141 7012246

[228] Wirt TC, McGee ZA, Oldfield EH, Meacham WF. 1979. Intraventricular administration of amikacin for complicated Gram-negative meningitis and ventriculitis. J Neurosurg 50:95–99. doi:10.3171/jns.1979.50.1.0095 363982

